# BCAFL: a secure and efficient blockchain framework for asynchronous federated learning

**DOI:** 10.1038/s41598-026-57780-z

**Published:** 2026-06-16

**Authors:** Jian Yun, Tao Liu

**Affiliations:** Dalian Minzu University, College of Computer Science and Engineering, Dalian, 116600 China

**Keywords:** Blockchain, Federated learning, Mutual information, Incentive mechanism, Information systems and information technology, Science, technology and society

## Abstract

Traditional synchronous Federated Learning (FL) is subject to the waiting latency inherent to synchronization mechanisms. Consequently, its convergence rate is constrained by straggler nodes within heterogeneous environments. Asynchronous Federated Learning (AFL) improves execution efficiency by removing global synchronization barriers. However, when integrated with blockchain for decentralized deployment, it still encounters challenges such as on-chain storage overhead arising from model parameters, convergence perturbations induced by stale gradients, and Byzantine security threats. To this end, this paper proposes BCAFL, a decentralized blockchain framework tailored for semi-asynchronous federated learning. BCAFL utilizes the InterPlanetary File System (IPFS) to implement off-chain storage for global model parameters. By integrating Model-Agnostic Meta-Learning (MAML) and PowerSGD, the framework enhances the model’s local adaptation capability on non-IID data while concurrently reducing communication overhead. To safeguard model security and convergence stability in asynchronous environments, this study develops a Mutual Information and Delay-Aware (MIDA) dynamic aggregation mechanism. This mechanism leverages Mutual Information (MI) to perform model verification for defense against poisoning attacks, while simultaneously modulating aggregation weights via a dynamic aggregation factor to effectively mitigate model oscillations inherent in asynchronous convergence. Additionally, this study develops a dynamic stake-based Verifiable Random Function (VRF) committee consensus mechanism. By quantifying election weights based on node contributions, this approach enhances consensus efficiency and resistance to Sybil attacks. Simulation results demonstrate that, compared with various existing baseline schemes, BCAFL maintains the convergence accuracy of the global model while reducing communication overhead. It effectively suppresses convergence oscillations caused by asynchronous delays and defends against poisoning and Byzantine attacks. Furthermore, when the network scale is expanded to 300 nodes, the consensus latency does not show a significant increase.

## Introduction

In recent years, Deep Learning (DL) technology has made substantial progress, leading to its extensive application across domains such as computer vision, natural language processing, and autonomous systems. The exceptional performance of DL models is largely attributed to their deep exploitation of large-scale, high-quality training data. Currently, distributed data sources, such as mobile terminals, private databases, and Internet of Things (IoT) sensors, contain vast volumes of valuable data^1^. However, since data movement is frequently constrained by bandwidth limitations and stringent privacy regulations, collecting these raw data poses inevitable communication and security challenges. To address these issues, Federated Learning (FL) has emerged as a promising solution, enabling multiple clients to build a joint learning model without exposing their private training data^2^. Orchestrated by a central server, clients periodically transmit their local updates to the server and retrieve the aggregated global model for the next round of training, until the model converges^3^.

However, traditional federated learning assumes that participants are honest, which is unrealistic. Malicious clients may send poisoned updates to manipulate model behavior, and research indicates that federated learning is highly susceptible to poisoning attacks^4^. Even the presence of a small number of malicious clients can significantly degrade global performance^5^. In addition, the centralized mode renders the system vulnerable to single points of failure or server outages^6^. Furthermore, synchronous federated learning cannot effectively utilize the resources of participating devices, causing devices with high computational capabilities to remain idle while waiting for other nodes to complete their local training. Although Asynchronous Federated Learning (AFL) and its evolved semi-asynchronous architectures better utilize the resources of participating devices, they also introduce challenges regarding gradient staleness and convergence stability^7^.

With its decentralized, tamper-proof, and auditable distributed ledger characteristics, blockchain provides an innovative technical path to solve the lack of trust and single-point-of-failure risks in FL^8^. Introducing blockchain to achieve decentralized model aggregation not only enhances the transparency of the collaborative learning process but also ensures the full traceability of training proofs^9^. However, existing blockchain FL systems still face multiple bottlenecks in practical deployment. First, the full-node deployment architecture^10^ requires all nodes to synchronize the complete ledger, leading to the rapid expansion of on-chain storage space and causing severe bandwidth overload; while schemes attempting to alleviate storage pressure by periodically deleting historical blocks^11^ inevitably undermine the security and credibility of the system. Second, although introducing a committee mechanism or dedicated verification nodes can effectively resist consensus attacks from malicious nodes^12^, in dynamic semi-asynchronous aggregation scenarios, stale gradients often exacerbate the convergence instability of the global model. Finally, although smart contract schemes based on public platforms such as Ethereum^13^ possess strong universality, directly executing complex anomaly detection and aggregation algorithms on-chain often faces severe performance bottlenecks. Therefore, there is currently a lack of an efficient and secure blockchain FL system that supports asynchronous aggregation and can defend against malicious attacks.

To address the aforementioned challenges, this paper proposes BCAFL, a blockchain framework tailored for semi-asynchronous federated learning. In terms of system architecture, this framework utilizes a distributed ledger to eliminate single-point-of-failure risks and introduces InterPlanetary File System (IPFS) to implement off-chain storage of model parameters, thereby alleviating on-chain storage expansion. During the local training phase, an optimization mechanism combining Model-Agnostic Meta-Learning (MAML) and PowerSGD is constructed to enhance the model’s local adaptation capability to non-independent and identically distributed (non-IID) data while effectively compressing the communication load. During the global aggregation phase, the Mutual Information and Delay-Aware (MIDA) dynamic aggregation mechanism is designed; it defends against poisoning attacks through mutual information filtering, and introduces a dynamic aggregation factor to alleviate aggregation deviation caused by data heterogeneity by quantifying the statistical consistency of gradients. Simultaneously, it suppresses asynchronous convergence oscillations caused by stale gradients based on the degree of round lag. During the consensus verification phase, a Verifiable Random Function (VRF) committee consensus mechanism based on dynamic stake is constructed to quantify node contributions into election weights, thereby enhancing the consensus efficiency and Sybil attack resistance capability of the system.

The main contributions of this paper are summarized as follows:

- Constructing a blockchain semi-asynchronous federated learning architecture. By introducing IPFS to implement off-chain storage of model gradients, only the hash index of the global model is recorded on-chain, thereby reducing the communication overhead of block propagation and alleviating the storage pressure of the blockchain.
- Designing a communication-efficient training mechanism combining MAML and PowerSGD. This mechanism utilizes PowerSGD to reduce communication overhead, and leverages MAML to enhance the model’s local adaptation capability to non-IID data, thereby ensuring the convergence accuracy of the global model in heterogeneous environments.
- Proposing the MIDA dynamic aggregation mechanism. This mechanism utilizes mutual information detection to eliminate poisoned updates, and constructs a dynamic aggregation factor to reduce the weights of highly deviated updates while preserving data feature diversity, and attenuates lagging parameters based on model staleness to suppress asynchronous oscillations.
- Constructing a VRF committee consensus mechanism based on dynamic stake. This mechanism updates dynamic stakes by quantifying node contributions, and uses this stake as the input weight for non-interactive committee elections, which enhances consensus efficiency and Sybil attack resistance capability while maintaining the operation of the decentralized system.
- Conducting simulation experiment evaluations. Results on the CIFAR10 and FEMNIST datasets show that BCAFL outperforms existing baseline schemes in convergence accuracy and stability when dealing with data heterogeneity, node lag, and poisoning attacks, and can maintain convergence stability under the interference of 1/3 Byzantine nodes; meanwhile, the framework effectively controls block expansion caused by continuous iterations, and maintains system resource consumption within a reasonable range under a network scale of 300 nodes, verifying its scalability.

The remainder of this paper is organized as follows: The “Related work” section introduces related work, and the  “Preliminaries” section introduces preliminaries. Section “The Proposed BCAFL framework” elaborates on the framework design, and the “Experimental evaluation” section conducts the experimental evaluation. The “Discussions and limitations” section discusses the limitations of the framework and future research directions, and the “Conclusion” section concludes the paper.

## Related work

The training efficiency of federated learning is inherently limited by frequent high-dimensional parameter exchanges among distributed clients^3^. To alleviate this bottleneck, academia has widely explored gradient compression techniques mainly based on sparsification and quantization^14^. Among them, SignSGD has become an efficient communication optimization scheme by extracting the sign bits of stochastic gradients^15^. As implemented by BASS, these signs are further encapsulated into compact bit-packets through bit-packing technology, achieving a reduction in transmission load^12^. However, SignSGD discards the numerical magnitude of the gradient during the quantization process. Although this scheme performs well in IID environments, the lack of magnitude information leads to limited convergence accuracy when handling highly non-IID data^16^. In contrast, PowerSGD^17^ adopts a low-rank decomposition strategy. As stated in the research of Vogels et al., this method maintains the structural integrity of the gradient matrix by capturing its main characteristic subspaces, thereby outperforming traditional element-wise quantization schemes in model approximation quality. In addition, MAML meta-learning is introduced into federated learning to achieve rapid local adaptation^18^. Inspired by this, the BCAFL framework proposed in this paper combines PowerSGD and MAML, enhancing the generalization capability of the model on heterogeneous data while reducing communication overhead.

The centralized aggregation of traditional federated learning is prone to triggering single points of failure and trust crises^8^. To establish a decentralized and trusted collaborative mechanism, existing studies have widely introduced blockchain technology, utilizing peer-to-peer networks to achieve decentralized model coordination and proofs recording. For example, the Biscotti framework proposed by Shayan et al.^19^ and the study by Kalapaaking et al.^20^ both verified the feasibility of utilizing distributed ledgers to record local updates and conduct cross-node auditing. However, defending against malicious poisoning attacks in decentralized networks still faces severe challenges. Existing on-chain verification mechanisms mainly rely on cross-validation, such as Blockflow^21^, or robust aggregation based on Euclidean distance, such as the Multi-krum algorithm adopted by SPDL^22^. The former is prone to causing additional privacy leakage and is limited by the quality of the validation set, while the latter is not only susceptible to communication uncertainties in decentralized scenarios, but also, when facing heterogeneous data distributions, leads to limited resistance against malicious nodes due to the difficulty in distinguishing between normal deviations and malicious poisoning^23^. In addition, the BSAFL^24^ framework constructs a node evaluation and weight allocation mechanism based on cross-entropy to resist abnormal updates; however, in non-IID scenarios, such loss-value-based measurement methods are difficult to accurately quantify the statistical heterogeneity of local data, causing it to be unable to effectively distinguish between normal data heterogeneous deviations and malicious parameter tampering. In architectures combining gradient compression, although SignSGD adopted by BASS^12^ can effectively limit the magnitude tampering of abnormal gradients through a node-summarized sign aggregation algorithm, in non-IID environments, due to the lack of gradient magnitude information, its majority voting mechanism makes it difficult to accurately identify sign perturbations when facing malicious poisoning attacks. Mutual Information (MI), as an information-theoretic metric, has been introduced into decentralized model verification mechanisms^25^. Unlike geometric metrics relying on Euclidean distance, it focuses on quantifying the degree of statistical dependence between different model parameters^23^. Even if local updates lose features due to low-rank approximation, the MI-based anomaly detection protocol can still identify statistical anomalies introduced by poisoning nodes by evaluating the mutual information gain among updates.

In federated learning, the heterogeneity of client computational resources and communication bandwidth causes the straggler node problem, which in turn affects the convergence efficiency of the global model. To address this issue, DAG-AFL^26^ adopts a Directed Acyclic Graph (DAG) topology, utilizing asynchronous concurrency mechanisms to alleviate waiting latency and directly merging the terminal nodes of the topology when generating the global model; AFLChain^27^ designs an asynchronous federated learning architecture based on a consortium blockchain, evaluating node reliability by introducing a reputation mechanism based on entropy weight. However, while pursuing high asynchronous concurrency, these two schemes sacrifice the detection capability for abnormal updates, causing both to be unable to effectively identify poisoned parameters in asynchronous and non-IID environments. BASS^12^ constructs a blockchain-based asynchronous SignSGD architecture, but this protocol relies on static sign consistency verification and does not introduce a gradient staleness adjustment mechanism, easily triggering convergence oscillations of the global model under heterogeneous networks. Meanwhile, AFLChain’s design of directly putting model parameters on-chain increases the storage overhead of nodes, limiting the scalability of the system. To alleviate such storage bottlenecks, decoupled storage architectures based on IPFS^28^ are widely explored. This architecture stores model parameters off-chain and records only lightweight Content Identifiers (CIDs) on-chain, effectively circumventing the ledger size explosion caused by frequent iterations.

The preceding analysis indicates that existing decentralized frameworks have made effective explorations in aspects such as model compression, topology improvement, and node behavior evaluation. However, under the chain architecture, existing schemes still exhibit limitations when confronting issues such as data heterogeneity caused by non-IID data, convergence oscillations triggered by stale gradients, model parameter poisoning attacks, and ledger storage expansion. To this end, this paper proposes BCAFL, a blockchain framework oriented towards semi-asynchronous FL, aiming to comprehensively address the aforementioned systemic challenges.

## Preliminaries

This section introduces the fundamental concepts of blockchain, IPFS, and VRF.

### Blockchain technology

Blockchain is a distributed ledger technology based on peer-to-peer networks, which can provide tamper-proof transaction records in trustless environments. Existing mainstream blockchain systems mostly adopt the Proof of Work (PoW) consensus mechanism, but this mechanism allows the existence of forks and has high confirmation latency, making it difficult to support high-frequency data interaction scenarios^29^. In contrast, Byzantine Fault Tolerance (BFT)-type consensus protocols possess built-in block finality and higher transaction throughput^30,31^. Therefore, the BCAFL framework proposed in this paper adopts a similar BFT consensus protocol to coordinate network nodes. BCAFL utilizes a distributed blockchain network to replace the traditional centralized server to execute the aggregation and update of the global model, aiming to eliminate single points of failure and ensure the security and credibility of the model aggregation and update process.

### IPFS

IPFS is a peer-to-peer distributed file system based on content addressing. It directly maps file content to a globally unique hash identifier (CID), and any minute data tampering will cause the hash value to change, thereby naturally guaranteeing the integrity of the stored files^28^. To overcome the storage bottleneck caused by directly putting global model parameters on-chain, we use IPFS to store global model parameters off-chain, and only record their corresponding CIDs on the blockchain, thereby reducing the storage cost of the blockchain and ensuring the integrity of the global model.

### VRF

A VRF is a cryptographic primitive that allows a private key holder to generate an output value that is both unpredictable and verifiable, along with its corresponding proof, based on a specific input. A verifier holding the corresponding public key can use the input, output value, and proof to verify that the output value is uniquely and correctly generated by the designated private key^32^. Combining this unique property, we use the dynamic stake accumulated by nodes within the system as the input weight to construct a VRF committee consensus mechanism based on dynamic stake.

## The Proposed BCAFL framework

We propose the BCAFL framework, aiming to achieve the following five core objectives: 1) eliminate the single-point-of-failure risk of traditional federated learning, and prevent malicious tampering during the model aggregation process; 2) under the premise of ensuring the convergence accuracy of the global model, effectively reduce the communication overhead caused by high-dimensional parameter interactions; 3) effectively suppress stale gradient oscillations in asynchronous environments, and mitigate the impact of poisoning attacks from potential malicious clients; 4) improve the generalization performance of the model in non-IID data scenarios, and enhance local adaptation efficiency; 5) maintain a lightweight ledger, and alleviate the ledger storage bottleneck caused by directly putting model parameters on-chain.

The security premises and attacker capability assumptions of this system are defined as follows:

- Regarding data poisoning (such as label-flipping) and model poisoning (such as bit-flipping) attacks during the local training phase, we assume that malicious clients in the framework are static and non-colluding. Specifically, malicious clients only execute fixed attack strategies and do not perform adaptive adjustments based on the state of the global model; meanwhile, they act independently of each other, do not possess knowledge of the framework’s detection rules, and lack the capability to collaboratively construct adversarial updates to evade detection.
- Regarding Byzantine behaviors during the blockchain consensus phase, such as malicious vetoing or injecting random noise, in order to clarify the defense boundaries of the framework, we refer to the theoretical fault-tolerance standard of traditional Byzantine Fault Tolerance (BFT) protocols, which requires that the total number of network nodes *N* and the number of Byzantine nodes *f* satisfy *N ≥ 3f + 1*. This paper assumes that the proportion of Byzantine nodes in the entire network does not exceed 1/3. This setting serves as the fundamental premise for subsequent framework design, theoretical convergence analysis, and simulation experiments.
- In terms of system architecture, we assume that federated learning clients and blockchain nodes remain independent in their roles and functions, with no overlap. Among them, clients only serve as transaction initiators responsible for local training, while blockchain nodes independently undertake the tasks of distributed ledger maintenance and consensus verification.

**Fig. 1:**
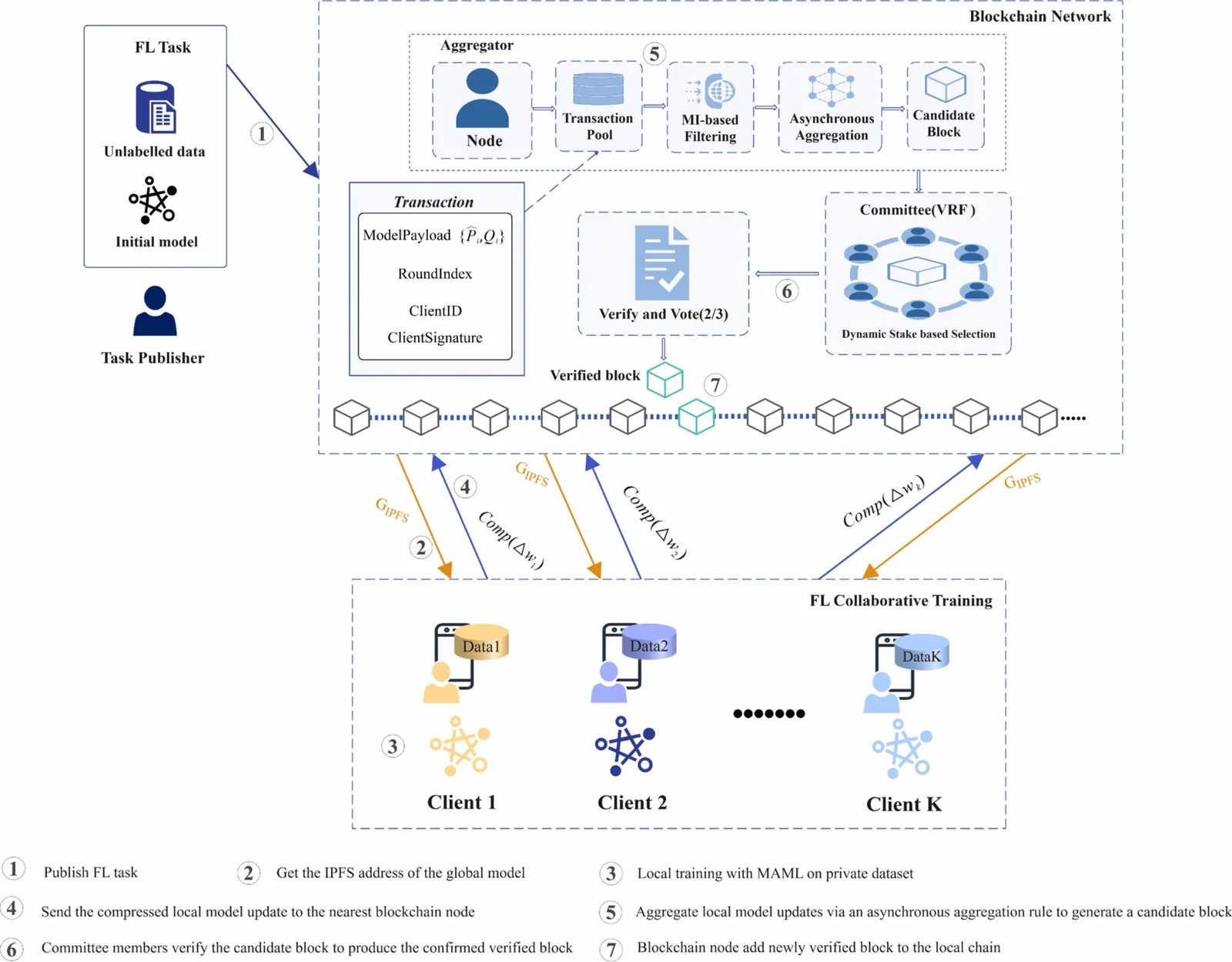
The system overview of BCAFL.

Within the aforementioned framework settings and security boundaries, BCAFL utilizes a decentralized blockchain system as the underlying coordination network. As illustrated in Fig. 1, the complete federated learning workflow operates according to the following steps:

- **Step 1**: The task publisher broadcasts the federated learning task, which encompasses unlabeled data and the initial global model stored in IPFS, to the blockchain network.
- **Step 2**: Blockchain nodes maintain replicas of the dataset. Clients retrieve the global model’s CID by querying active neighboring nodes and subsequently download the model parameters from IPFS.
- **Step 3**: Starting from the global model, clients execute training iterations based on MAML utilizing local private data.
- **Step 4**: Upon completion of local training, clients utilize PowerSGD to compress the model updates and transmit the compressed gradients, $\text {Comp}(\Delta w)$, alongside their digital signatures as transactions to the nearest nodes.
- **Step 5**: Upon completing signature and dimensional verification for the received transactions, nodes broadcast them to the transaction pool for asynchronous caching. When the valid transaction volume reaches the threshold *M*, nodes trigger the MIDA mechanism. This mechanism initially detects model gradients via mutual information and subsequently employs a dynamic aggregation factor to perform semi-asynchronous aggregation on the remaining updates, thereby mitigating the impact of stale gradients generated by heterogeneous nodes on model stability. After the aggregation process is completed, nodes store the generated global model $w^{t+1}$ in IPFS and construct candidate blocks.
- **Step 6**: The candidate blocks are dispatched to the consensus committee, which is elected based on node dynamic stakes and the VRF algorithm. Committee members are responsible for validating the correctness of the global model calculation. If a candidate block collects more than 2/3 of the approval votes, it is formally confirmed and broadcast across the network.
- **Step 7**: Each node appends the validated block to its local chain. The system allocates rewards or deducts stakes based on node behavior. The updated node stakes will serve as weight parameters for the VRF election in the subsequent round.

### Initialization

The initialization phase of BCAFL primarily involves the launch of the permissioned blockchain and the configuration of node states. This paper assumes that a trusted authority bootstraps the system to launch the permissioned blockchain and admit qualified blockchain nodes. During the registration phase, the system assigns each blockchain node *n* a pair of VRF keys $(SK_n, PK_n)$ for dynamic committee election, a key pair $(sk_n, pk_n)$ for identity authentication and transaction signing, and a default initial stake $S_n^0$ for the incentive mechanism. The genesis block of the blockchain is a fixed block that contains the system’s initial configuration parameters and the foundational state declarations of the nodes. Once the underlying network is ready, the task publisher releases a federated learning task to the system. This task transaction encapsulates the unlabeled dataset $D_u$ utilized for MIDA model verification, the IPFS hash index $CID^0$ of the initial global model $W^0$, and the various hyperparameters required for the federated learning task.

### Semi-asynchronous aggregation and block validation

**Fig. 2:**
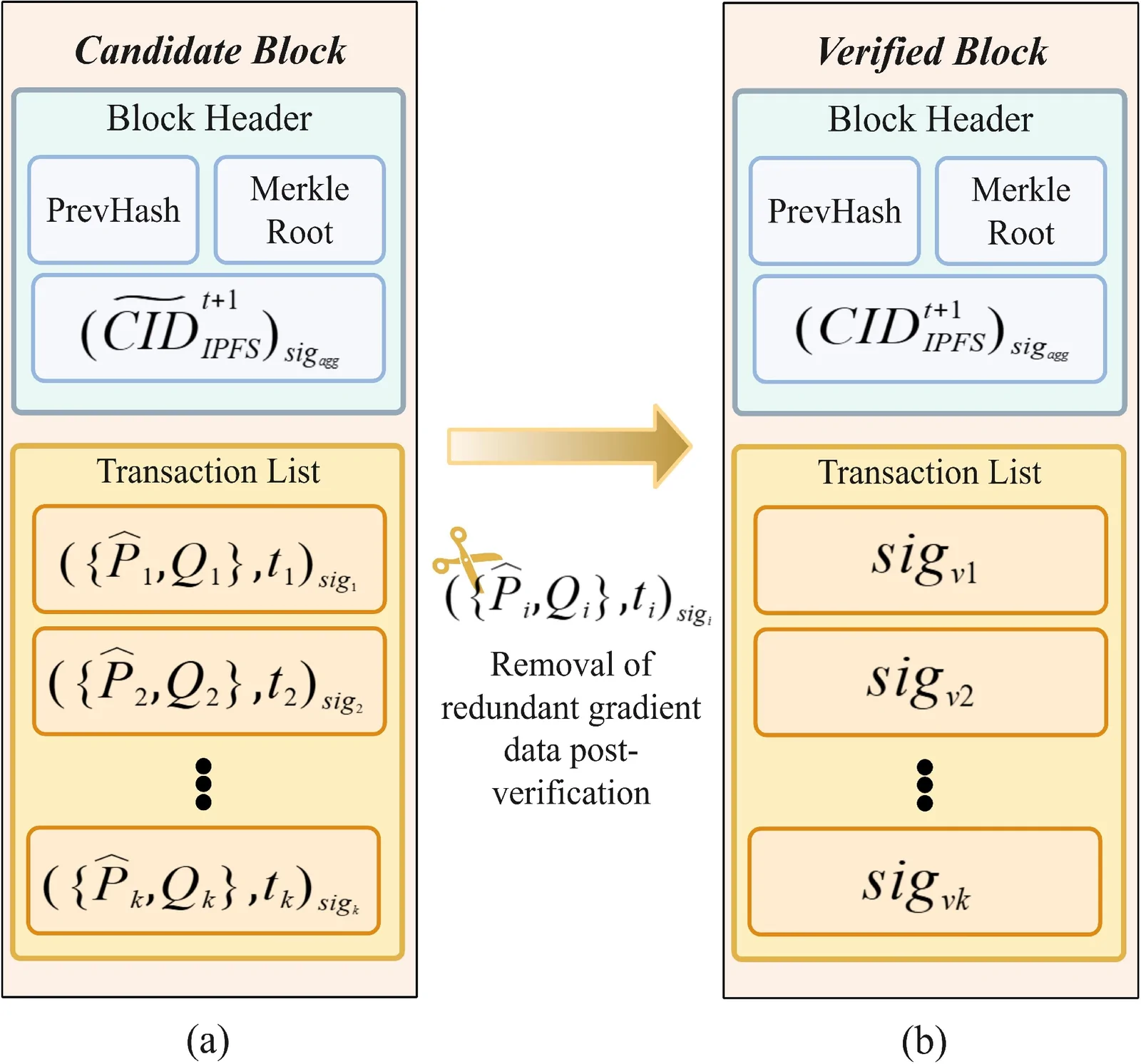
Data structures of candidate and verified blocks.

After the FL task is released, BCAFL adopts a blockchain-based semi-asynchronous FL architecture to execute model iterations. By combining non-blocking asynchronous training at the client level with threshold-triggered aggregation at the node level, it effectively balances the execution efficiency of the framework and the convergence stability of the global model. At the client level, each device independently executes local training without waiting for a global synchronization barrier; after client *i* completes its computation, it encapsulates the PowerSGD-compressed low-rank matrix pair $\{\hat{P}, Q\}$, the training round $Round_t$, the public key $ID_i$, and the signature $Sig_i$ into a $Tx_{update}$ transaction and broadcasts it to the network. This non-blocking asynchronous upload mechanism effectively alleviates the waiting latency caused by straggler nodes in traditional synchronous FL. At the blockchain node level, to avoid model oscillations caused by individual updates in pure asynchronous modes, the framework introduces a threshold-based non-blocking caching mechanism. Nodes temporarily store the received update transactions in the transaction pool. When the number of collected valid $Tx_{update}$ transactions reaches the preset threshold $M_{req}$, nodes terminate the waiting period for the current round and trigger the MIDA dynamic aggregation mechanism. The MIDA mechanism not only filters the batch of model gradients via mutual information but also adaptively adjusts the weights of stale gradients through a dynamic aggregation factor, thereby effectively smoothing convergence perturbations in the asynchronous environment. After the robust aggregation by MIDA generates the global model $w^{t+1}$, nodes store it in the IPFS network and construct candidate blocks, as illustrated in Fig. 2a.

Unlike traditional frameworks that rely on centralized servers, BCAFL clearly distinguishes the independent functions of FL clients and blockchain nodes, achieving logical decoupling at the functional level. This decentralized architectural design provides a reliable execution guarantee for the semi-asynchronous workflow. Specifically, the underlying consensus protocol explicitly defines the legitimacy criteria for candidate blocks, and the verification process is executed by the committee elected via Algorithm 3. During the consensus phase, committee members extract the transactions within the block and re-execute the aggregation logic to validate the correctness of the global model computation. If an aggregation node attempts to trigger aggregation prematurely when the number of transactions within the block is less than the preset threshold $M_{req}$, or if it packs invalid signatures, the candidate block will be directly rejected for violating the consensus criteria. According to the PBFT protocol, a candidate block must collect more than 2/3 of valid committee signatures to achieve consensus. Therefore, through a distributed committee verification mechanism, the blockchain ensures the strict execution of semi-asynchronous aggregation rules in a trustless manner. After consensus is reached, the gradient transaction data within the candidate block is deleted and replaced with the committee’s consensus signatures, ultimately transforming into a verified block and completing the on-chain process, as illustrated in Fig. 2b.

### Communication-efficient local training based on MAML and PowerSGD

Unlike obtaining the global model from a centralized server, BCAFL allows clients to acquire the latest global model by sending requests to the blockchain network. Blockchain nodes return the IPFS address of the latest global model $w^t$ and its recorded training round, based on which clients verify whether the global model has been updated. Leveraging the content-addressing feature of IPFS, clients can download the global model. To reduce communication overhead in the decentralized blockchain network and improve model performance under non-IID data distributions, this section proposes a local training mechanism that combines MAML with PowerSGD.

During the *t*-th FL round, client *i* receives the global model $w^t$ and utilizes it as the initial state for local training, i.e., $w_{i,t}^0 = w^t$. To adapt to heterogeneous data distributions, the client executes *ι* steps of local meta-updates. For the *k*-th local update step ($k \in \{1, 2, \dots , \iota \}$), the client initially utilizes the local data batch $D_i^k$ to execute a single-step gradient descent for fast adaptation, obtaining the temporary personalized model $\tilde{w}_{i,t}^k$, as formulated in Eq. (1):

1 $$\begin{aligned} \tilde{w}_{i,t}^k = w_{i,t}^{k-1} - \alpha \nabla f_i(w_{i,t}^{k-1}; D_i^k) \end{aligned}$$

where *α* is the inner-loop step size of meta-learning. Subsequently, the client utilizes an independent data batch $D_i'^k$ to compute the outer-loop meta-gradient. To reduce the computational overhead of second-order derivatives, this paper employs the first-order approximation FO-MAML ^18^ to estimate the meta-gradient, as formulated in Eq. (2):

2 $$\begin{aligned} \nabla F_i(w_{i,t}^{k-1}) \approx \nabla f_i(\tilde{w}_{i,t}^k; D_i'^k) \end{aligned}$$

Based on this first-order meta-gradient, the client performs a local meta-update on the current initial parameters, as formulated in Eq. (3), where *β* is the outer-loop step size:

3 $$\begin{aligned} w_{i,t}^k = w_{i,t}^{k-1} - \beta \nabla F_i(w_{i,t}^{k-1}) \end{aligned}$$

After *ι* meta-update steps, the local model $w_{i,t}^\iota$ is obtained. To reduce communication overhead, the client encapsulates the current parameter update $\Delta \theta = w_{i,t}^\iota - w^t$ with the compression residual from the previous round to construct the target compression vector *Δ w*. PowerSGD low-rank decomposition is applied to *Δ w*, reconstructing the gradient vector *Δ w* into multiple parameter matrices $\{U_1, \dots , U_W\}$, where each $U \in \mathbb {R}^{n \times m}$ is decomposed into $\hat{P} \in \mathbb {R}^{n \times r}$ and $Q \in \mathbb {R}^{m \times r}$. The client encapsulates the compressed low-rank matrices $\hat{P}$ and *Q* into a transaction for submission to the blockchain; the specific workflow is shown in Algorithm 1.

**Algorithm 1:**
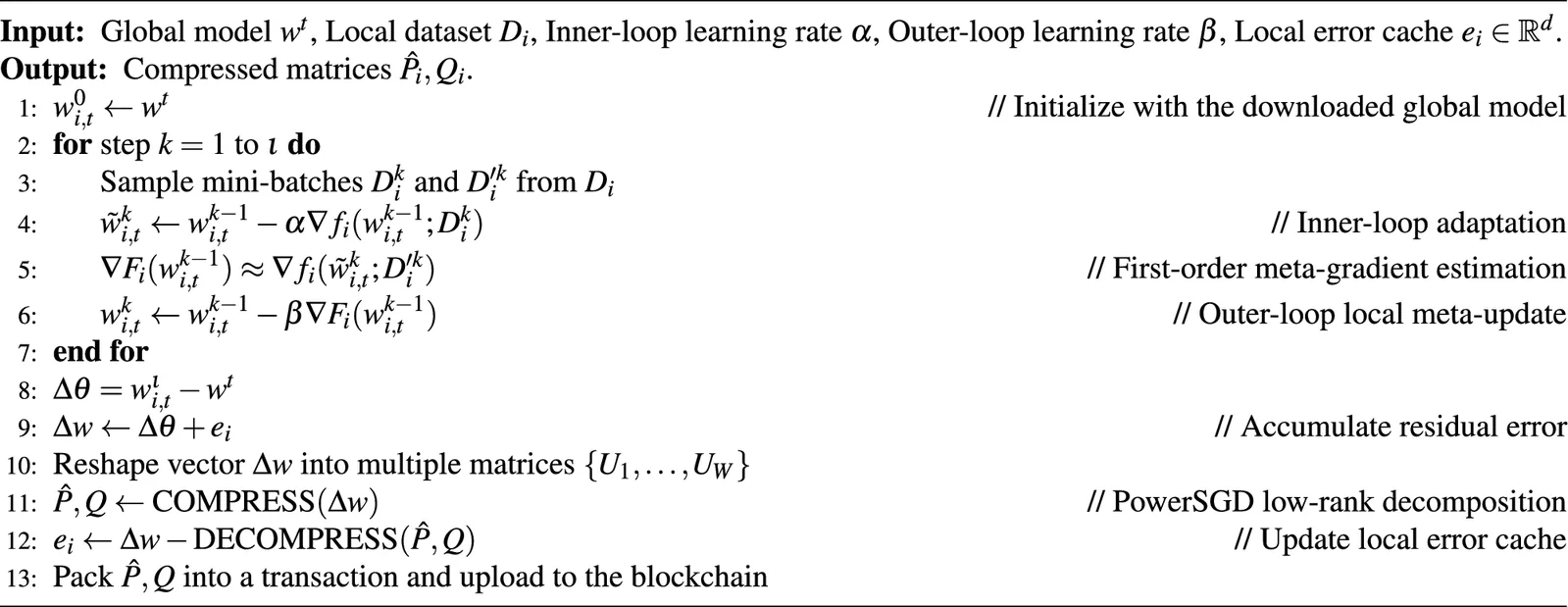
Local Update via MAML and PowerSGD

Furthermore, although the threat model in this paper primarily focuses on poisoning and Byzantine attacks, the introduction of the PowerSGD low-rank decomposition mechanism, while reducing communication overhead, objectively increases the difficulty of feature inference attacks such as Deep Leakage from Gradients (DLG). Such attacks typically rely on intercepting complete and precise gradient vectors to execute reverse optimization. In the communication process of this framework, however, the network transmits the dimension-reduced low-rank matrix pair $\{\hat{P}, Q\}$ rather than the complete local update vector *Δ w*. This low-rank approximation process is inherently an irreversible lossy compression, which prevents attackers from losslessly recovering the original gradients even if they intercept $\{\hat{P}, Q\}$. Therefore, although this framework does not introduce specific cryptographic defense mechanisms against inference attacks, the communication dimensionality reduction process objectively restricts the reverse derivation targeting private data, thereby providing parameter inputs with a certain degree of anti-inference capability for the subsequent semi-asynchronous aggregation at the node level.

**Fig. 3:**
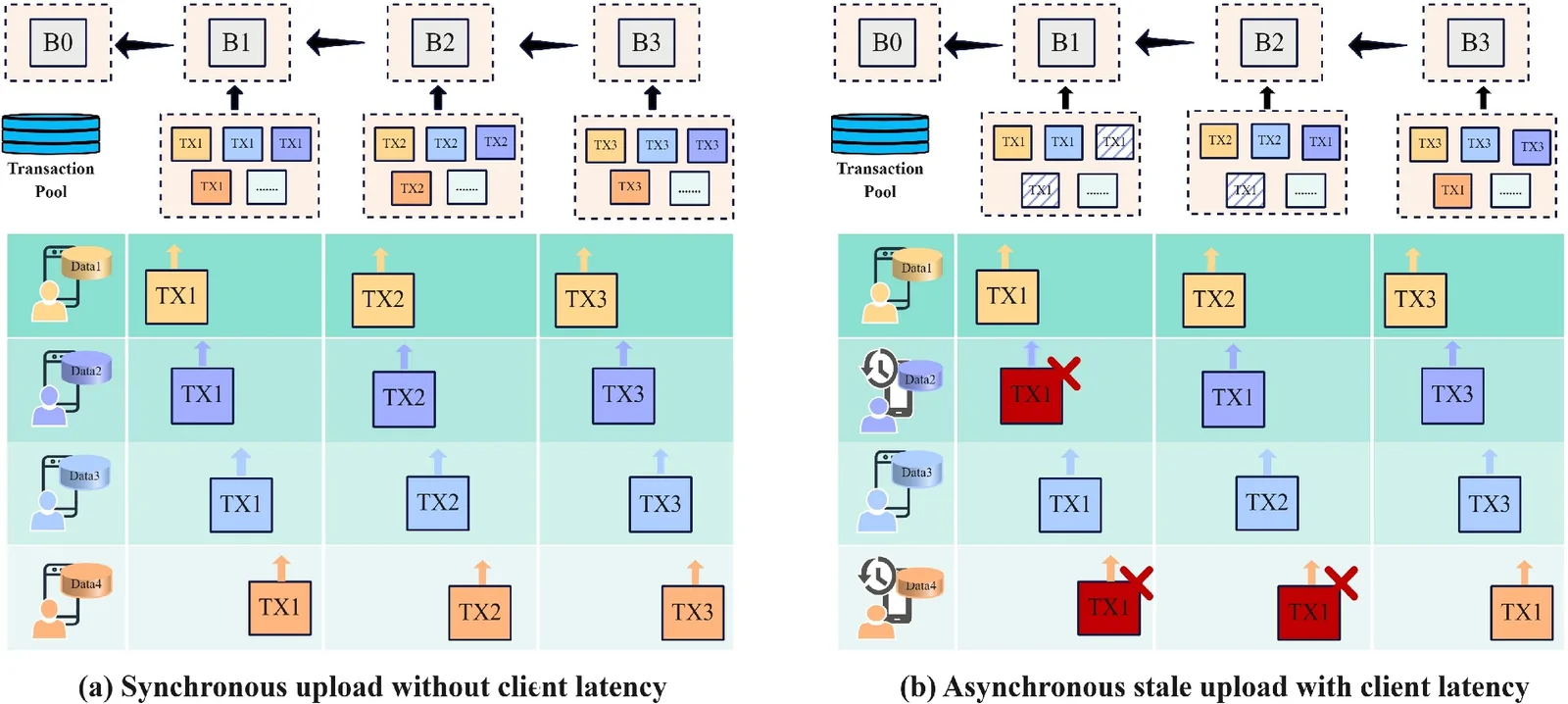
Comparison of synchronous and semi-asynchronous aggregation workflows.

### Mutual information and delay-aware dynamic aggregation

In the semi-asynchronous architecture, affected by the heterogeneity of node computational power and fluctuations in network bandwidth, a significant asynchronous lag exists between the local training of FL clients and global aggregation. In an ideal environment, model updates are executed based on the synchronous round parameter alignment depicted in Fig. 3a, whereas straggling clients in practical operations introduce stale gradients, as illustrated in Fig. 3b. Directly aggregating such lagging parameters can induce oscillations in the global model during the convergence process, and even lead to training divergence.

To constrain the negative interference of model gradients and defend against potential poisoning attacks, when a node receives update transactions $Tx_{update}$ and reaches the preset threshold $M_{req}$, it triggers the MIDA dynamic aggregation mechanism, as shown in Algorithm 2. In specific execution, to suppress potential poisoning attacks, MIDA adopts an MI-based model filtering mechanism ^25^. It identifies anomalous updates by measuring the correlation between the outputs of client models. Let $D_u$ be a globally shared unlabeled dataset. For any pair of clients (*i*, *j*), the Pearson correlation coefficient $\rho _{i,j}$ of their local models’ output vectors on $D_u$ is first calculated, based on which the mutual information is computed as formulated in Eq. (4):

4 $$\begin{aligned} MI_{i,j} = -\frac{1}{2} \log (1 - \rho _{i,j}^2) \end{aligned}$$

Subsequently, the average mutual information $MI_i$ between each client and all other clients is computed as the MI score for that client. To achieve anomaly detection, twice the Median Absolute Deviation (MAD) is adopted as the filtering criterion. The filtering logic for the reliable update set *Z* is formulated in Eq. (5):

5 $$\begin{aligned} Z = \left\{ i \left| \frac{|MI_i - median(MI)|}{MI_{madn}} \le 2 \right. \right\} \end{aligned}$$

where *median*(*MI*) reflects the central tendency of the overall distribution, and $MI_{madn}$ is the normalized median absolute deviation used to measure the dispersion of the MI score distribution. When a client’s score falls outside the range of twice $MI_{madn}$, the node determines it as an anomalous update and rejects its participation in the current round of aggregation.

After obtaining the reliable update set *Z*, the aggregation node reads the local training round $\tau _i$ of the client from the transaction $Tx_{update}$. The staleness of this local update is defined as the difference between the current global iteration round *t* and the round $\tau _i$, i.e., $t - \tau _i$. To suppress convergence oscillations caused by model gradients, the node combines the mutual information score and the temporal decay term $e^{-\mu (t - \tau _i)}$ to calculate the dynamic aggregation factor $\gamma _i^t$ for each update in set *Z*, as formulated in Eq. (6):

6 $$\begin{aligned} \gamma _i^t = \text {Norm}(MI_i) \times e^{-\mu (t - \tau _i)} \end{aligned}$$

where *μ* is the decay hyperparameter, and the normalized mutual information score $\text {Norm}(MI_i)$ is utilized to quantify the statistical consistency of each gradient within the reliable update set *Z*. In non-IID environments, this mechanism reduces the aggregation proportion of highly deviated updates, maintaining the stability of the global convergence direction while preserving the diversity of data features. The temporal decay term $e^{-\mu (t - \tau _i)}$ is employed to control the aggregation proportion of cross-round stale gradients within the global model. In a semi-asynchronous environment, directly executing equal-weight aggregation on stale gradients interferes with the global update direction and induces convergence oscillations. Therefore, this mechanism adaptively decays the aggregation weights of outdated updates based on the round lag, ensuring that the latest local updates dominate the iteration direction of the global model. This effectively balances data diversity in heterogeneous networks while suppressing the negative interference of stale parameters, thereby enhancing convergence stability in the semi-asynchronous environment.

After computing the dynamic aggregation factor $\gamma _i^t$ for each client, to ensure the scale consistency of global gradients during semi-asynchronous aggregation and control model oscillations caused by stale gradients, the aggregation node executes an asynchronous update on the local model update amounts $\Delta w_i$ within set *Z* according to Eq. (7):

7 $$\begin{aligned} w^{t+1} = w^t + \eta \cdot \lambda _t \sum _{i \in Z} \hat{\gamma }_i^t \cdot \Delta w_i \end{aligned}$$

where *η* is the global learning rate, and $\Delta w_i$ is the local model update amount recovered via decompression by the client. $\hat{\gamma }_i^t$ is the locally normalized weight, defined as $\hat{\gamma }_i^t = \frac{\gamma _i^t}{\sum _{j \in Z} \gamma _j^t}$, which mitigates aggregation weight deviations caused by the dynamic fluctuation of the number of effectively participating nodes in a single round. $\lambda _t$ is the adaptive damping coefficient, defined as $\lambda _t = \frac{\sum _{i \in Z} \gamma _i^t}{1 + \sum _{i \in Z} \gamma _i^t}$. When the sum of effective update weights decreases due to high data heterogeneity or severe network latency, $\lambda _t$ attenuates accordingly. By dynamically reducing the global update step size, it effectively suppresses the negative perturbations on global model convergence caused by highly deviated and stale parameters. Once the global model $w^{t+1}$ is computed, the node resets the current update cache pool and encapsulates $w^{t+1}$ into a candidate block, dispatching it to the committee members for verification.

**Algorithm 2:**
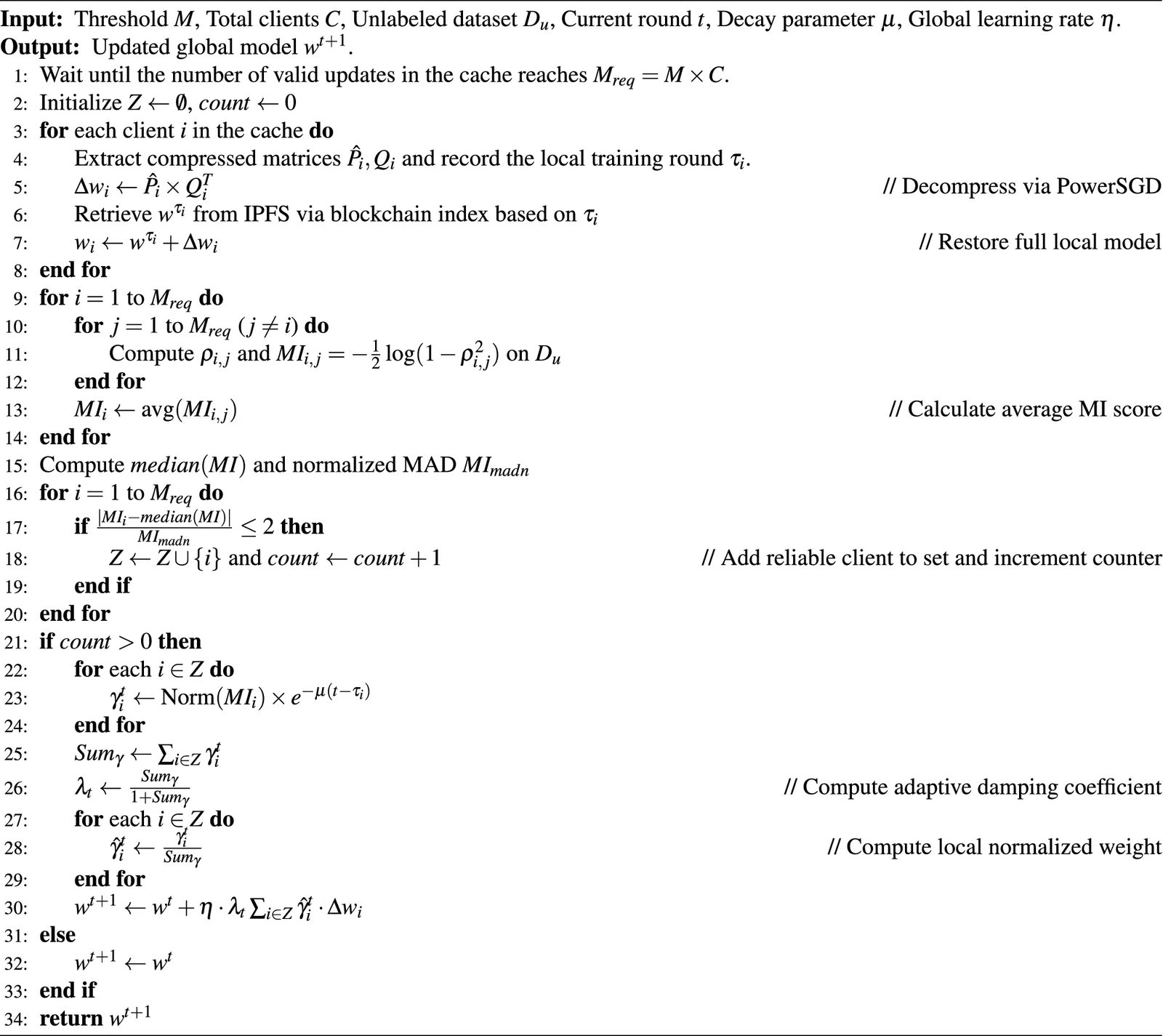
Mutual Information and Delay-Aware Dynamic Aggregation

### VRF committee consensus mechanism based on dynamic stake

To validate the legitimacy of candidate blocks, the framework needs to construct a decentralized consensus committee. During this process, blockchain nodes incur distinct computational and communication overheads when executing tasks such as model verification, parameter aggregation, and block consensus. To evaluate the actual participation of nodes, this paper proposes a dynamic stake mechanism capable of adaptively updating a node’s stake value based on its role state and specific contributions during the consensus process. When a node is elected as an aggregation node, its primary task is to execute global model aggregation and generate candidate blocks. To prevent high-stake nodes from long-term monopolization of block generation rights, the reward for this node is strictly limited to a fixed-percentage commission extracted from the committee. In the *t*-th consensus round, the dynamic stake update formula for node *n* is:

8 $$\begin{aligned} S_n^{t+1} = S_n^t + R_{base} + \frac{\sigma }{K} \sum _{l \in V} \Phi (v_l^t) \end{aligned}$$

In Eq. (8), $R_{base}$ is the base reward for nodes appending blocks to the local ledger to maintain consistency, $\sigma \in (0, 1)$ is the base commission coefficient, *K* is the committee size for the current round, *V* is the set of committee members for the current round, and $\Phi (v_l^t)$ is the verification contribution value of member *l* used to quantify the computational expenditure of the node, as shown in Eq. (9):

9 $$\begin{aligned} \Phi (v_l^t) = R_{base} \cdot (1 - e^{-b \alpha _v}) \end{aligned}$$

where *b* is the number of local models contained in the candidate block, and $\alpha _v$ serves as a tuning factor to control the growth rate of verification rewards. Committee members execute model verification and state consistency checks according to security protocols, and their stake updates are shown in Eq. (10). Other nodes only receive the base reward $R_{base}$ to maintain consistency.

10 $$\begin{aligned} S_n^{t+1} = S_n^t + R_{base} + (1 - \sigma )\Phi (v_l^t) \end{aligned}$$

If a node exhibits non-compliant behavior, its reward for the current round is $R_n = 0$, and a slashing operation is executed on its stake. The stake update rule for a non-compliant node is $S_n^{t+1} = \max (0, S_n^t - Pen_n)$, where the penalty amount $Pen_n$ is defined as follows:

11 $$\begin{aligned} Pen_n = {\left\{ \begin{array}{ll} S_n^t \cdot (1 - \omega ^{H_n}), & \text {malicious attack behavior} \\ \varphi R_{base}, & \text {negative response behavior} \end{array}\right. } \end{aligned}$$

In Eq. (11), $H_n$ is the cumulative number of malicious attacks, and $\omega \in (0, 1)$ is the penalty attenuation base, ensuring that the penalty severity escalates with the number of violations while reserving a fault-tolerance margin for occasional verification deviations. Malicious attack behaviors include data tampering, malicious signatures, or deliberately vetoing legitimate blocks during audits; $\varphi \in (0, 1)$ is the negative response penalty factor, determining the fixed deduction from the base reward for passive violations. Negative responses include network timeouts or abnormal disconnections.

After determining the dynamic stake of nodes, BCAFL needs to construct a consensus committee to execute block verification. Inspired by BEFL ^28^, this paper adopts a VRF committee election scheme to ensure the non-interactivity and Sybil resistance characteristics of decentralized governance. In this scheme, dynamic stakes are directly used as the weight input for the election, binding the node’s election probability to the quantification of its actual contributions.

In the *(t+1)*-th consensus round, node *n* uses its dynamic stake $S_n^{t+1}$ as the weight input and utilizes the random seed $seed_t$ from the previous block to execute Algorithm 3. The node first calculates a pseudo-random hash value $hash_n$ and its corresponding proof $\pi _n$ via its private key $SK_n$:

12 $$\begin{aligned} hash_n, \pi _n = \text {VRF}_{SK_n}(seed_t) \end{aligned}$$

The hash value is mapped to yield a random value $rand_n = \frac{hash_n}{2^{hashlen}}$ uniformly distributed within the interval [0, 1). The judgment condition and probability $p_n$ for node *n* to be elected as a committee member are defined as:

13 $$\begin{aligned} p_n = \Pr \left( rand_n < \min \left( 1, \frac{c K S_n^{t+1}}{S_{total}^{t+1}}\right) \right) = \min \left( 1, \frac{c K S_n^{t+1}}{S_{total}^{t+1}}\right) \end{aligned}$$

In Eq. (13), $S_{total}^{t+1}$ is the total network stake, *K* is the target size, and *c > 1* is the amplification factor. Let the random variable $X = \sum _{n \in N} X_n$ denote the total number of elected individuals in the whole network, with its expected value *E(X) = cK*. Since the process of each node using its local private key to execute the VRF lottery does not interfere with one another, this process can be viewed as a series of independent Bernoulli trials. Based on this statistical property, the failure probability that the candidate nodes are less than *K* can be derived from the Chernoff bound. Let $\varepsilon = \frac{c-1}{c}$, then the failure probability satisfies:

14 $$\begin{aligned} \Pr (X \le K) \le e^{-\frac{K(c-1)^2}{2c}} \end{aligned}$$

This inequality indicates that by reasonably setting the amplification factor *c*, BCAFL can guarantee that the consensus committee has a sufficient number of members with an exponentially converging probability.

**Algorithm 3:**
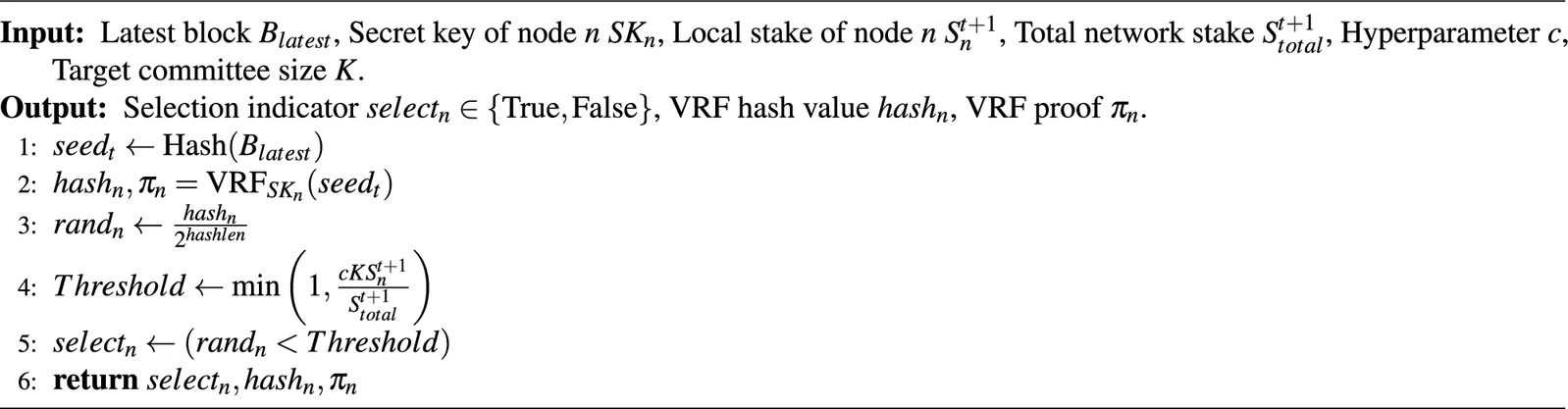
Committee Member Selection

Nodes satisfying the condition $select_n = \text {True}$ broadcast the proofs $\{hash_n, \pi _n\}$ to the network. Other nodes extract the public parameters and execute verification utilizing the corresponding public keys. Once the network collects *K* compliant members, the committee construction phase for the current round concludes, and the first *K* nodes to obtain network confirmation collectively constitute the consensus committee for the current round. Through the consensus verification of the committee, the verified block is ultimately appended to the underlying ledger, thereby completing the global model iteration and state update for the current round.

### Convergence analysis

To verify the theoretical validity of the framework, this section rigorously analyzes the convergence of BCAFL under conditions of non-IID data, PowerSGD compression, semi-asynchronous latency, and Byzantine node interference. Let the global loss function be $F(w) = \frac{1}{C} \sum _{i=1}^C F_i(w)$. The fundamental assumptions for the theoretical analysis are defined as follows:

#### Assumption 1

*(Smoothness and Lower Bound):* *F*(*w*) is an *L*-smooth function and possesses a global lower bound $F^*$.

#### Assumption 2

*(Bounded Gradients and Heterogeneous Gradient Deviation):* The expected squared norm of the local stochastic gradients is bounded, i.e., $\mathbb {E}\left[ \Vert \nabla F_i(w)\Vert ^2\right] \le G^2$. The expected squared norm of the global aggregated gradients is bounded, i.e., $\mathbb {E}\left[ \Vert g^t\Vert ^2\right] \le \tilde{G}^2$. Influenced by the non-IID data distribution, the expected deviation between the local true gradient and the global gradient is bounded, satisfying $\mathbb {E}\left[ \Vert \nabla F_i(w) - \nabla F(w)\Vert ^2\right] \le \kappa ^2$.

#### Assumption 3

*(Compression Error):* The PowerSGD operator $\text {Comp}(\cdot )$ possesses a constant $\delta \in (0, 1]$ that satisfies the expected compression error $\mathbb {E}\left[ \Vert \text {Comp}(x) - x\Vert ^2\right] \le (1 - \delta )\Vert x\Vert ^2$.

#### Assumption 4

*(Asynchronous Latency):* The maximum latency between the current round *t* and the round $\tau _i$ at which the stale gradient was generated is bounded, i.e., $t - \tau _i \le \tau _{\max }$.

#### Assumption 5

*(Byzantine Interference):* Referring to the theoretical fault tolerance upper bound of the BFT protocol, we assume that the proportion of Byzantine nodes in the network does not exceed 1/3. Under this assumption, after being filtered by the MIDA mechanism, the expected squared norm of the residual malicious gradient deviation in the reliable set *Z* satisfies $\mathbb {E}\left[ \Vert \Delta _{Byz}\Vert ^2\right] \le \epsilon _{Byz}^2$.

In the *t*-th round, the global update rule is $w^{t+1} = w^t - \eta _t g^t$, where $\eta _t = \eta \cdot \lambda _t$ (*η* is the global learning rate, and $\lambda _t = \frac{\sum _{i \in Z} \gamma _i^t}{1 + \sum _{i \in Z} \gamma _i^t}$ is the adaptive damping coefficient). The actual aggregated expected gradient $g^t$ is formulated as:

15 $$\begin{aligned} g^t = \sum _{i \in Z} \hat{\gamma }_i^t \text {Comp}(\nabla F_i(w^{\tau _i})) + \Delta _{Byz} \end{aligned}$$

where $\hat{\gamma }_i^t = \frac{\gamma _i^t}{\sum _{j \in Z} \gamma _j^t}$ is the normalized weight. Assuming the damping coefficient has a positive lower bound $\lambda _{\min } > 0$, then it holds that $\eta \lambda _{\min } \le \eta _t \le \eta$.

#### Theorem 1

Under the established assumptions, if the global learning rate satisfies $\eta \le \frac{1}{2L}$, then the expected squared norm of the global gradient of BCAFL after *T* iteration rounds satisfies:

16 $$\begin{aligned} \frac{1}{T} \sum _{t=0}^{T-1} \mathbb {E}\left[ \Vert \nabla F(w^t)\Vert ^2\right] \le \frac{2(F(w^0) - F^*)}{\eta \lambda _{\min } T} + \frac{L \eta \tilde{G}^2}{\lambda _{\min }} + \frac{4}{\lambda _{\min }} \left( (1 - \delta )G^2 + L^2 \eta ^2 \tau _{\max }^2 \tilde{G}^2 + \kappa ^2 + \epsilon _{Byz}^2 \right) \end{aligned}$$

#### Proof

Based on the *L*-smoothness, by performing a Taylor expansion of $F(w^{t+1})$ at $w^t$ and taking the expectation, we obtain:

17 $$\begin{aligned} \mathbb {E}[F(w^{t+1})] \le F(w^t) - \eta _t \langle \nabla F(w^t), \mathbb {E}[g^t] \rangle + \frac{L \eta _t^2}{2} \mathbb {E}\left[ \Vert g^t\Vert ^2\right] \end{aligned}$$

*□*

Applying the inner product identity and Young’s inequality to process the cross term $-\eta _t \langle \nabla F(w^t), \mathbb {E}[g^t] \rangle$, and combining it with the global gradient upper bound $\tilde{G}^2$ from Assumption 2, we obtain:

18 $$\begin{aligned} \mathbb {E}[F(w^{t+1})] \le F(w^t) - \frac{\eta _t}{2} \Vert \nabla F(w^t)\Vert ^2 + \frac{\eta _t}{2} \mathbb {E}\left[ \Vert g^t - \nabla F(w^t)\Vert ^2\right] + \frac{L \eta _t^2}{2} \tilde{G}^2 \end{aligned}$$

Regarding the error term $\mathbb {E}\left[ \Vert g^t - \nabla F(w^t)\Vert ^2\right]$, we expand and decompose it as follows:

19 $$\begin{aligned} g^t - \nabla F(w^t) = \sum _{i \in Z} \hat{\gamma }_i^t \left[ \text {Comp}(\nabla F_i(w^{\tau _i})) - \nabla F_i(w^{\tau _i}) + \nabla F_i(w^{\tau _i}) - \nabla F_i(w^t) + \nabla F_i(w^t) - \nabla F(w^t) \right] + \Delta _{Byz} \end{aligned}$$

Utilizing the inequality $\Vert a+b+c+d\Vert ^2 \le 4(\Vert a\Vert ^2+\Vert b\Vert ^2+\Vert c\Vert ^2+\Vert d\Vert ^2)$ and the convex combination property of $\sum _{i \in Z} \hat{\gamma }_i^t$, the four error terms are respectively bounded as follows:

- **Compression error:** Based on the local gradient upper bound $G^2$ from Assumption 2 and Assumption 3, this is bounded by $4(1-\delta )G^2$.
- **Latency error:** Derived from the accumulation of parameter updates, we have $\Vert w^{\tau _i} - w^t\Vert \le \eta \tau _{\max } \tilde{G}$. Subsequently, utilizing the *L*-smoothness yields $\Vert \nabla F_i(w^{\tau _i}) - \nabla F_i(w^t)\Vert \le L\Vert w^{\tau _i} - w^t\Vert$. After squaring, this term is bounded by $4L^2 \eta ^2 \tau _{\max }^2 \tilde{G}^2$.
- **Heterogeneous gradient deviation:** According to Assumption 2, this is bounded by $4\kappa ^2$.
- **Byzantine interference:** Constrained by the MIDA boundary, according to Assumption 5, this is bounded by $4\epsilon _{Byz}^2$.

Substituting the aforementioned results back into the Taylor expansion, and summing over *t = 0, … , T-1*, to extract $\frac{1}{T} \sum _{t=0}^{T-1} \mathbb {E}\left[ \Vert \nabla F(w^t)\Vert ^2\right]$, we bound the left side of the equation using $\eta _t \ge \eta \lambda _{\min }$, while taking the upper bound for the right side with $\eta _t \le \eta$. Consolidating the constant terms yields Theorem 1.

#### Corollary 1

Based on Theorem 1, if the learning rate is selected as $\eta = \mathcal {O}(1/\sqrt{T})$, then both the optimization error term and the gradient variance term on the right side of the equation are bounded by $\mathcal {O}(1/\sqrt{T})$. At this point, $\frac{1}{T} \sum _{t=0}^{T-1} \mathbb {E}\left[ \Vert \nabla F(w^t)\Vert ^2\right] \le \mathcal {O}(1/\sqrt{T}) + \mathcal {O}(1)$, indicating that BCAFL can converge at a sub-linear rate to a bounded error neighborhood.

Theorem 1 and Corollary 1 clearly demonstrate that the convergence error bound of BCAFL is composed of multiple terms. Among them, the first term is the optimization error that decays as the number of iteration rounds *T* increases; the remaining terms strictly bound the system perturbations caused by the PowerSGD compression ratio *δ*, the gradient deviation induced by data heterogeneity $\kappa ^2$, the maximum asynchronous latency $\tau _{\max }$, and the Byzantine filtering residual $\epsilon _{Byz}$. This theoretically confirms the robustness of the proposed framework in non-ideal environments.

## Experimental evaluation

The experimental environment of this framework was built on a server equipped with an NVIDIA RTX 3080 Ti GPU and an Intel(R) Xeon(R) Silver 4214R CPU @ 2.40GHz. We implemented the BCAFL framework using Go 1.16 and Python 3.7, where the distributed communication and blockchain network were developed based on Go, while model training and gradient computation were executed using Python and the PyTorch framework. The experiments were deployed in a local area network (LAN) environment. Specifically, blockchain nodes act as full nodes to maintain the ledger and execute consensus verification; FL clients only utilize local data to perform model training, interact with blockchain nodes by calling RPC interfaces to retrieve the IPFS hash recorded on the chain to download the global model, and upload the compressed gradients after completing the local update. This framework adopts a semi-asynchronous aggregation mechanism. In each iteration round, all clients concurrently execute local model training and asynchronously upload their updates. The blockchain nodes set the aggregation cache threshold to 60%. When a node collects model updates satisfying this threshold, it immediately triggers the MIDA mechanism to execute global model aggregation. Furthermore, we randomly sampled 1,000 samples from the test set and removed their labels to serve as the unlabeled dataset $D_u$ for mutual information computation. To ensure the reliability and validity of the experimental results, all experiments were independently repeated three times, and the average values were taken as the final performance metrics. During the local model training and meta-update phases, clients uniformly employed the Stochastic Gradient Descent (SGD) optimizer for parameter updates. Detailed experimental parameter configurations are presented in Table 1.

**Table 1:** The default parameters of BCAFL.

Parameter	Value
Number of blockchain nodes *N*	100
Committee size *K*	15
Aggregation cache threshold *M*	60%
Number of FL clients *C*	15
PowerSGD compression rank (FEMNIST)	2
PowerSGD compression rank (CIFAR10)	4
Local batch size *B*	64
Global learning rate *η*	1.0
Local training steps *ι*	5
Inner-loop learning rate *α*	0.01
Outer-loop learning rate *β*	0.001

The experimental evaluation employs two typical non-IID datasets: FEMNIST and CIFAR10. Among them, the FEMNIST dataset is preprocessed via the LEAF federated benchmarking tool ^33^, containing 62 classes of handwritten samples from over 3,500 authors. This experiment assigns handwritten samples from different authors to distinct clients, thereby constructing a non-IID scenario. The CIFAR10 dataset contains 10 classes of RGB images and adopts the partitioning method of LotteryFL to construct a non-IID distribution ^34^. We set the class imbalance degree for each client to 0.75, meaning that each client is assigned a specific dominant class, while the remaining data is composed of other randomly assigned minority classes. For the FEMNIST task, this experiment employs a Convolutional Neural Network (CNN) architecture comprising two convolutional layers, two pooling layers, and two fully connected layers; for the CIFAR10 task, the ResNet-14 architecture is selected ^35^.

To comprehensively evaluate the performance of BCAFL, this paper selects five baseline schemes for comparison: (1) FedAvg ^2^: a classical synchronous federated learning algorithm, serving as the foundational performance comparison baseline; (2) FedAsync ^36^: an asynchronous federated learning method that performs weighted aggregation of stale updates via a time decay function; (3) Multi-Krum ^37^: a robust aggregation algorithm based on Euclidean distance, which filters anomalous gradients and defends against malicious node poisoning by selecting a subset closest to the majority of updates; (4) BASS ^12^: a blockchain-based asynchronous SignSGD framework that achieves decentralized semi-asynchronous federated learning through sign compression and asynchronous aggregation thresholds; (5) BSAFL ^24^: a blockchain-based asynchronous federated learning framework that evaluates node model quality via cross-entropy and combines a time factor to adjust global aggregation weights. This paper selects BASS and BSAFL as decentralized baselines to quantitatively evaluate the comprehensive performance of BCAFL in coping with data heterogeneity, latency lag, and malicious attacks.

**Table 2:** Model Accuracy under various scenarios (%).

Scheme	CIFAR10			FEMNIST		
	No Attack	LF	BF	No Attack	LF	BF
FedAvg	60.9	55.3	46.2	75.1	70.5	55.9
FedAsync	56.9	50.9	39.0	76.4	65.2	43.1
Multi-Krum	62.1	59.4	53.2	76.6	73.5	70.0
BASS	52.1	42.6	40.9	73.5	68.4	68.8
BSAFL	57.8	52.1	49.7	74.6	72.7	71.9
BCAFL	66.5	62.3	60.1	83.3	77.8	80.2

### Model accuracy and security assessment

This section evaluates the convergence performance of BCAFL in an attack-free environment, and tests the security of the framework under malicious attacks through the test accuracy in poisoning attack scenarios, the recall and false positive rates of the mutual information anomaly detection mechanism, and the model convergence accuracy in a Byzantine environment.

Targeting static non-colluding poisoning attacks at the client level, we set the proportion of malicious clients to 20% and implement two representative poisoning attacks: Label-Flipping (LF) ^38^ and Bit-Flipping (BF) ^39^. Specifically, LF is a data poisoning attack where malicious clients substitute the true labels of training samples with target labels to induce misclassification in the global model. BF is a model poisoning attack where malicious clients directly undermine the weight update of the global model by uploading the scaled negation of their locally computed gradients. Table 2 summarizes the final accuracies of each algorithm under these diverse scenarios.

In the attack-free scenario, the model accuracies of BCAFL on the CIFAR10 and FEMNIST datasets reach 66.5% and 83.3%, respectively, achieving superior convergence precision compared to the baseline algorithms. Experimental data demonstrate that BCAFL employs the MAML mechanism to enhance the model’s adaptation capability to local heterogeneous data, and utilizes PowerSGD to preserve the primary structural features of the gradients while compressing communication overhead. This enables the framework to maintain high convergence accuracy in a non-IID environment. In contrast, although FedAsync can mitigate the stale gradient issue, it fails to correct the local update direction deviation caused by data heterogeneity in a non-IID environment, limiting its convergence accuracy. Multi-Krum relies on Euclidean distance to filter anomalous updates; however, in a non-IID scenario, it is prone to misjudging normal data distribution deviations as anomalous updates, causing the global model to lose partial heterogeneous data features. BASS compresses gradients through sign quantization, a mechanism that discards the precise numerical magnitude information of gradients. In a non-IID scenario, its capability to represent the magnitude of parameter updates is limited, leading to a decline in its convergence accuracy. BSAFL’s cross-entropy-based weight allocation strategy struggles to accurately quantify the statistical deviation of local data in a non-IID scenario, restricting the final convergence accuracy of the global model.

When facing poisoning attacks, all baseline algorithms exhibit varying degrees of performance degradation. Lacking an anomaly filtering mechanism, FedAvg and FedAsync directly incorporate poisoned parameters into the global update, resulting in a significant accuracy drop for the global model under BF attacks. The Euclidean distance metric relied upon by Multi-Krum struggles to distinguish between legitimate data distribution deviations and malicious poisoning perturbations in a non-IID environment, limiting its defensive effectiveness. The sign quantization mechanism of BASS suffers from quantization distortion due to the lack of gradient magnitude information, making its majority voting aggregation susceptible to malicious sign-flipping interference. The sensitivity of the cross-entropy-based evaluation metric in BSAFL is reduced under heterogeneous data distributions, rendering it unable to effectively defend against malicious tampering of local parameters. In contrast, BCAFL utilizes mutual information to measure the statistical consistency of parameter distributions, successfully distinguishing normal data heterogeneity from anomalous updates. It demonstrates superior robustness across various attack scenarios and achieves a higher final test accuracy than the baseline schemes.

To quantitatively analyze the contribution of the mutual information defense phase in the MIDA mechanism, this paper evaluates its detection performance under different attack scenarios, as shown in Table 3. Experimental results indicate that under LF and BF attacks, the recall of the defense mechanism on both datasets exceeds *93%*, with the corresponding False Negative Rate (FNR) ranging from *1.27%* to *6.06%*. This set of data demonstrates that the mutual information filtering mechanism can identify the vast majority of malicious models and effectively isolate them from the reliable update set. Furthermore, in a non-IID data environment, the False Positive Rate (FPR) of this mechanism ranges from *2.06%* to *6.01%*. The relatively low FPR metric reflects that the mutual information-based filtering mechanism can effectively distinguish between parameter manipulation caused by malicious attacks and gradient deviations resulting from normal data heterogeneity. By maintaining a low FPR, this mechanism filters malicious updates while preserving local data features, thereby providing secure and diverse parameter inputs for the subsequent aggregation phase.

**Fig. 4:**
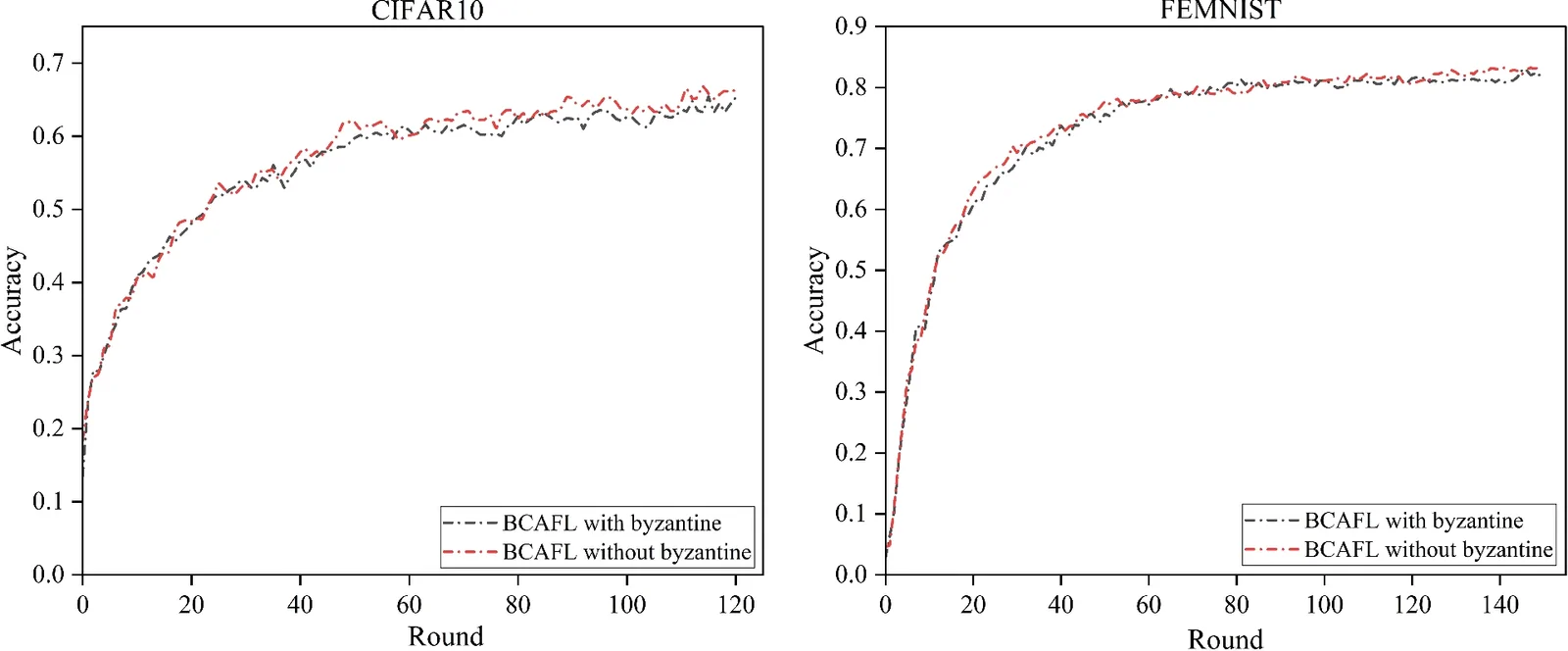
Convergence performance of BCAFL under Byzantine attacks.

Building upon the guaranteed security of local parameter inputs, this paper further evaluates the fault-tolerance performance of BCAFL against the Byzantine behavior of blockchain nodes. Referring to the theoretical fault-tolerance upper bound of the BFT protocol and the evaluation settings in existing literature ^28^, the proportion of Byzantine nodes in the network is set to 1/3 of the total nodes. In the experiment, Byzantine nodes participate in the committee election and candidate block generation processes, with their specific attack behaviors configured as follows: during the consensus verification phase, Byzantine nodes elected to the committee cast opposing votes against legitimate candidate blocks and approving votes for illegitimate ones; during the model aggregation phase, Byzantine nodes violate the secure semi-asynchronous aggregation protocol by constructing random noise following a standard normal distribution to replace the real local gradients for uploading, thereby executing a Gaussian attack. Fig. 4 compares the convergence performance of BCAFL without Byzantine attacks and under the aforementioned attacks. As illustrated, in both the CIFAR10 and FEMNIST tasks, even when facing Byzantine anomalies from 1/3 of the nodes, the convergence curves of BCAFL highly overlap with those in the attack-free scenario, and the test accuracy exhibits no significant decline.

**Table 3:** Performance of the MI-based filtering mechanism (%).

Dataset	Attack type	Precision	Recall	FPR	FNR
CIFAR10	LF	80.26	97.78	6.01	2.22
	BF	92.15	96.70	2.06	3.30
FEMNIST	LF	83.21	98.73	4.98	1.27
	BF	89.86	93.94	2.65	6.06

### Ablation study

To verify the necessity of each core component in the BCAFL framework, experiments were conducted on the non-IID CIFAR10 and FEMNIST datasets to compare the impacts of MAML and PowerSGD on model convergence accuracy, and to validate the security guarantees of the dynamic stake mechanism for the framework under attacks involving 30% Byzantine nodes.

**Fig. 5:**
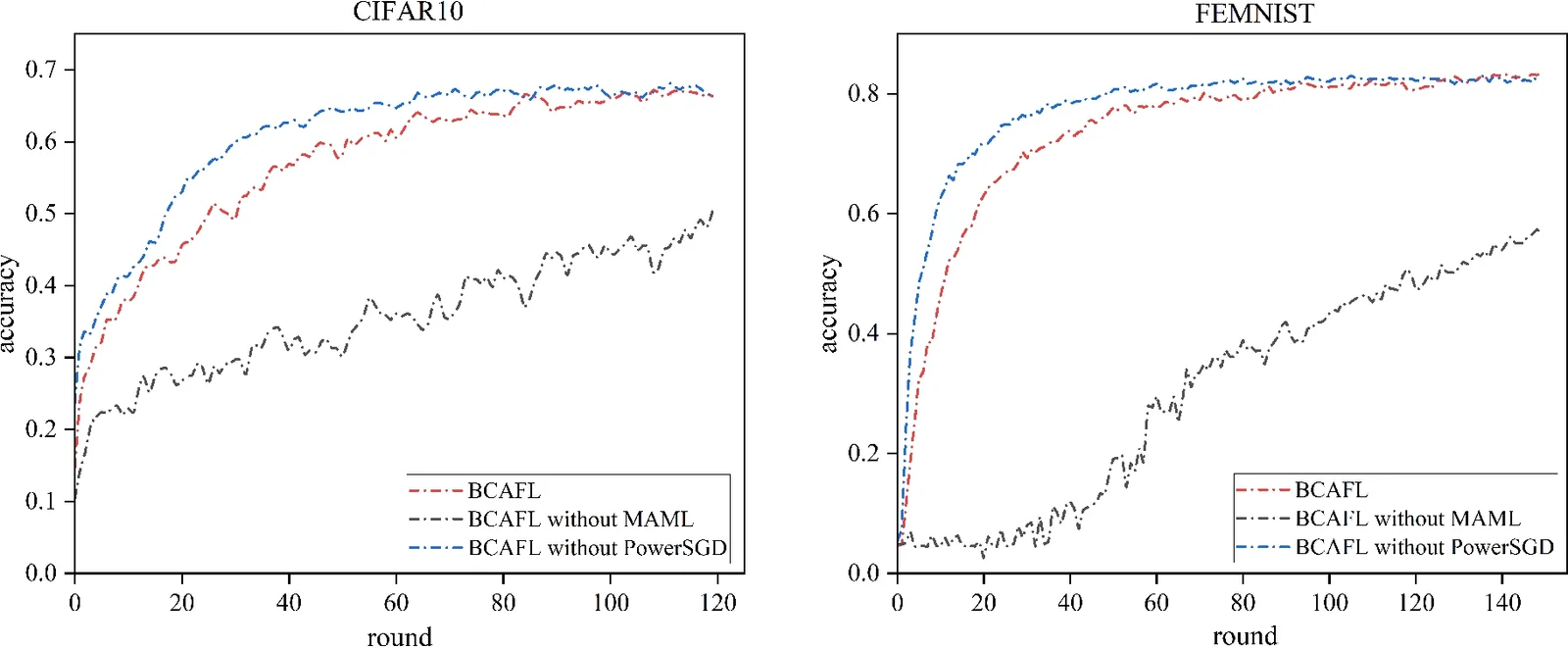
Impact of MAML and PowerSGD on model convergence.

Figure 5 illustrates the variations in model accuracy of BCAFL and its variants with MAML and PowerSGD removed, respectively. The MAML strategy adopted by BCAFL achieves local adaptation of the model to heterogeneous data distributions, enabling the test accuracy on CIFAR10 to reach 66.5%. In contrast, after removing the MAML mechanism, the model only converges to 50.4% under the same non-IID scenario. This performance difference is equally significant on the FEMNIST dataset. Regarding communication overhead, BCAFL introduces the PowerSGD mechanism to compress gradients to reduce the transmission payload. Compared to the uncompressed transmission scheme, although BCAFL exhibits relatively slower convergence in the early training stages due to compression errors, its final convergence accuracy remains essentially on par with the uncompressed scheme.

**Fig. 6:**
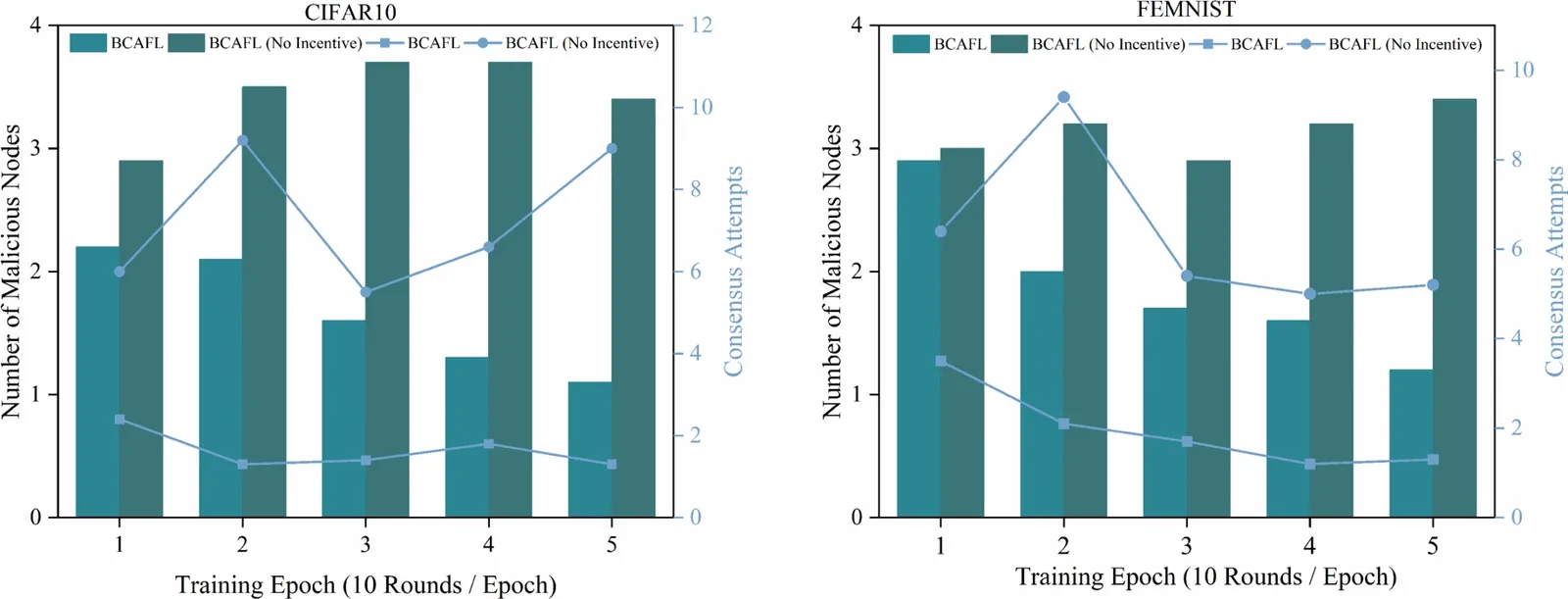
Ablation analysis of the dynamic stake mechanism.

To evaluate the impact of the dynamic incentive mechanism on framework security, Fig. 6 compares the variations in the number of malicious nodes in the consensus committee and the number of re-elections between BCAFL and BCAFL (No Incentive) over five consecutive cycles. The experiment sets the consensus committee size to *K = 15*, and the theoretical fault tolerance upper bound for a single-round committee to ensure consensus consistency is 4. In the dynamic incentive mechanism, the committee election probability $p_n$ of a node is positively correlated with its dynamic stake. When a node executes Byzantine behaviors such as tampering with data or malicious signing, a penalty mechanism is triggered to slash its stake. This continuous attenuation of stake weakens the competitiveness of malicious nodes in subsequent VRF elections, thereby effectively restricting them from entering the consensus committee. As shown by the experimental results in Fig. 6, due to the lack of stake penalty constraints, the average number of selected malicious nodes in the BCAFL (No Incentive) scheme fluctuates continuously near the failure threshold, placing consensus security in a critical state. Conversely, as the execution cycles progress, BCAFL successfully suppresses the average number of malicious nodes within the committee in the CIFAR10 and FEMNIST tasks to 1.1 and 1.2, respectively. Furthermore, the decline in the proportion of malicious nodes directly optimizes the consensus efficiency of the system. As honest nodes dominate the committee, the number of consensus attempts in BCAFL gradually decreases with the progression of cycles, converging to a near-ideal block generation state by the fifth cycle. In contrast, because the BCAFL (No Incentive) scheme cannot effectively block the interference of malicious nodes, it maintains high re-election redundancy throughout the entire operation period, generating uncontrollable consensus latency overhead.

### Convergence stability in asynchronous environments

In heterogeneous networks, disparities in node computational capabilities lead to gradient upload lags, which consequently induce convergence oscillations in the global model. To evaluate the framework’s robustness against asynchronous environments, this section employs model convergence accuracy and curve stability under different proportions of delayed nodes as evaluation metrics. The experiments set the proportions of delayed clients to 20% and 40%, respectively, to simulate gradient upload lags in heterogeneous network environments.

**Fig. 7:**
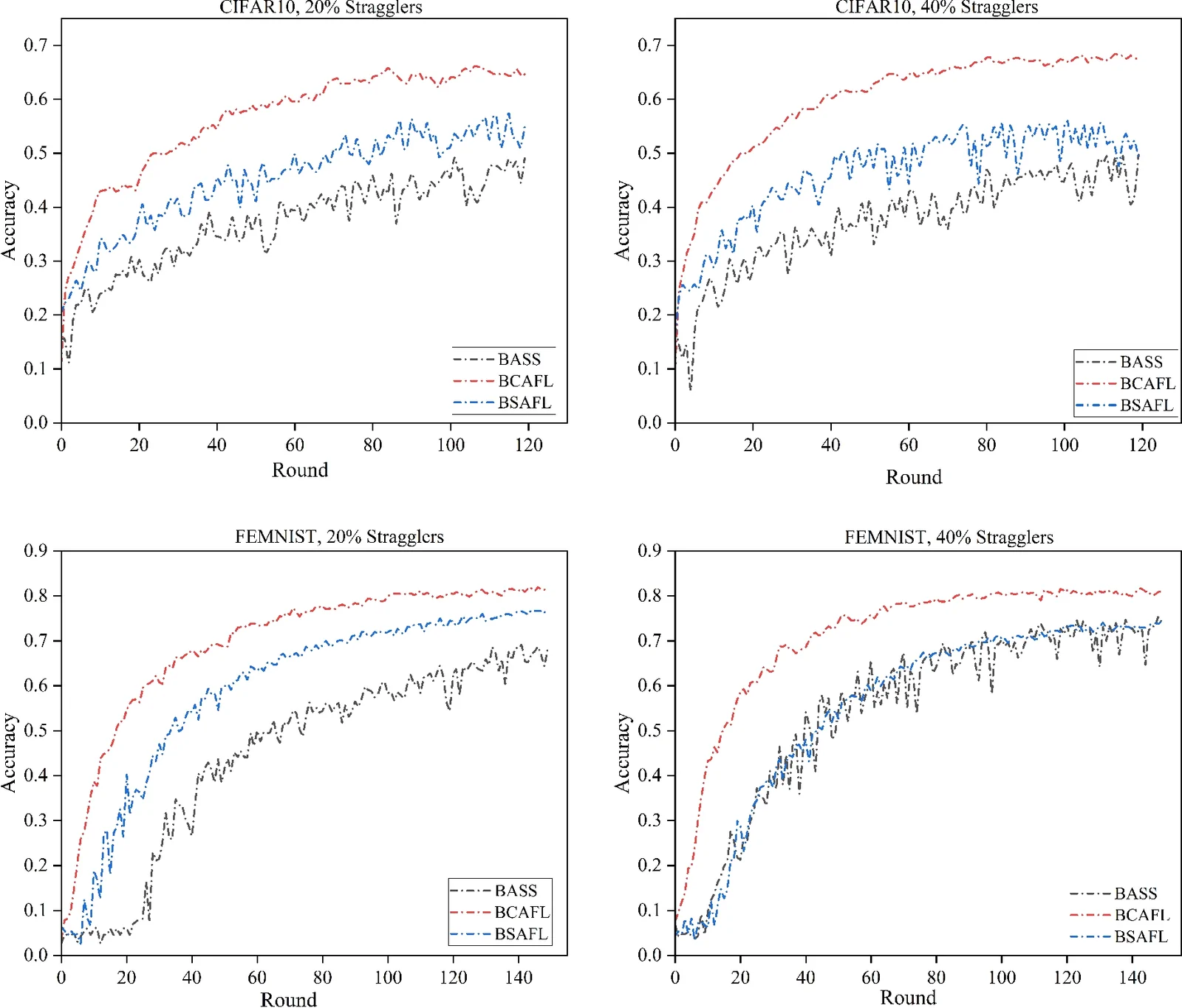
Comparison of convergence stability among BASS, BSAFL, and BCAFL under different straggler proportions in asynchronous environments.

Figure 7 illustrates the convergence curves of various algorithms under different proportions of delayed clients. As evidenced by Fig. 7, the sign aggregation mechanism adopted by BASS lacks a regulatory strategy for stale updates. Directly merging outdated local parameters into the global model induces a deviation in the update direction, causing the convergence curve of this algorithm to exhibit instability across all test scenarios. Although BSAFL adjusts the aggregation weights by incorporating a time factor, the metric it relies on struggles to accurately quantify the validity of stale gradients under heterogeneous data distributions. This causes the algorithm to still exhibit convergence fluctuations on the CIFAR10 dataset; on the FEMNIST dataset, while its convergence curve is relatively smooth, the final precision is limited, and its overall accuracy is lower than that of BCAFL. In contrast, BCAFL utilizes the MIDA dynamic aggregation mechanism to adaptively implement weight decay on stale updates. While ensuring the data participation of nodes, it mitigates the perturbations of stale parameters on the global model, enabling the framework to maintain a steady convergence process under varying delay proportions and achieve the highest accuracy.

**Fig. 8:**
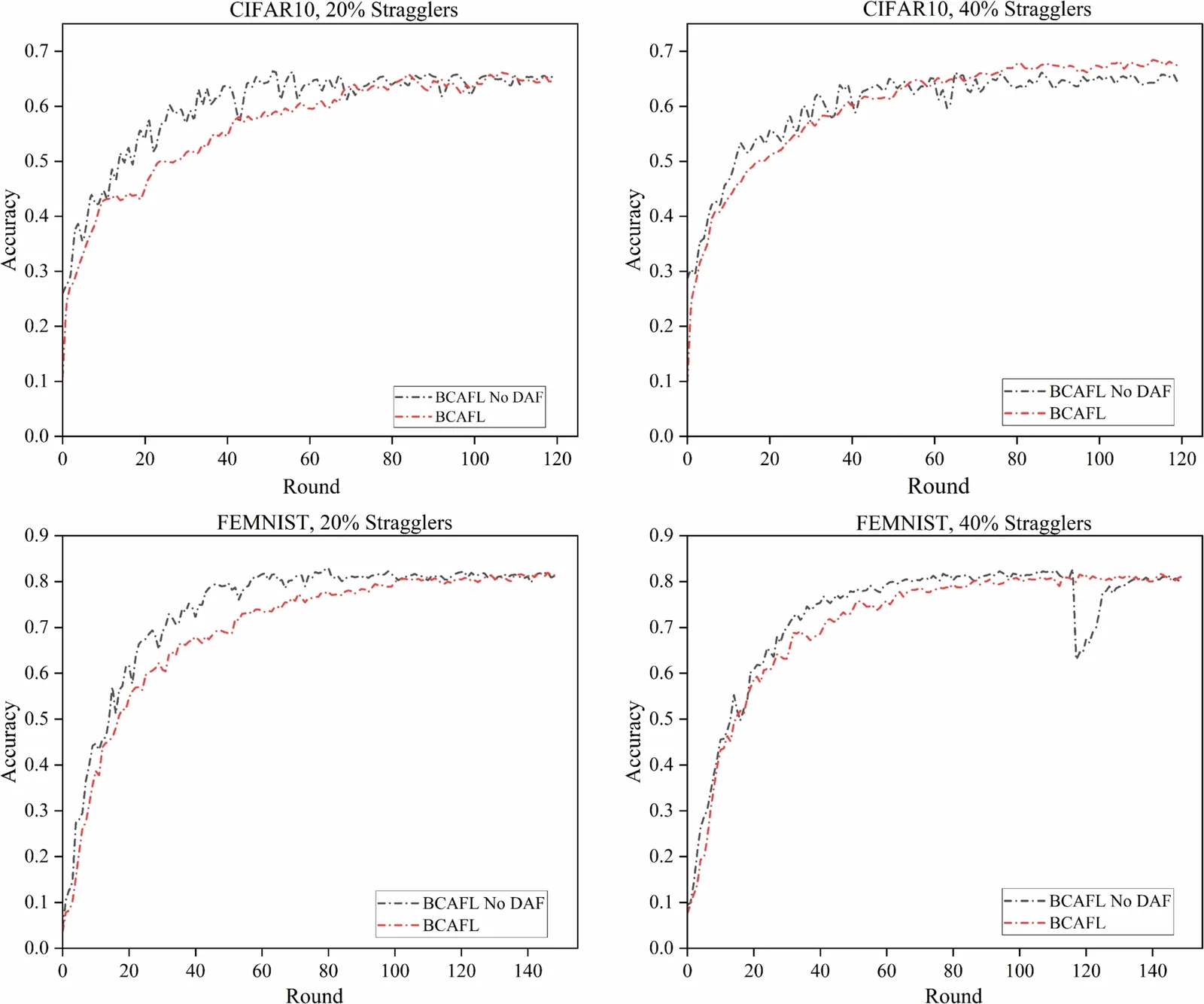
Ablation study of the dynamic aggregation factor under different delayed client proportions.

We further analyze the contribution of the dynamic aggregation factor within the MIDA mechanism to convergence stability and dissect the specific role of the delay-aware mechanism therein. Figure 8 compares the performance curves of BCAFL and the variant with the dynamic aggregation factor removed (BCAFL No DAF) under different proportions of delayed clients. Experimental results indicate that, due to the lack of adaptive regulation capabilities for data heterogeneity and gradient staleness, the scheme without this factor enforces equal-weight aggregation for all local updates. This leads to persistent fluctuations in the accuracy curves of this scheme on both datasets. Notably, in the FEMNIST task with a 40% delayed client proportion, this scheme experiences an accuracy drop around the $120^{\text {th}}$ round. In contrast, BCAFL can adaptively regulate the aggregation weights via the dynamic aggregation factor, mitigating the interference of heterogeneous data and stale parameters on global optimization, thereby ensuring steady model convergence.

Building upon validating the overall effectiveness of the dynamic aggregation factor, this experiment further evaluates the robustness of the BCAFL framework in complex network environments with intensified asynchrony. By comparing the convergence performance of the BCAFL framework under the 20% and 40% delay proportion scenarios, it can be observed that, under the premise of maintaining a constant degree of data heterogeneity, as the network delay proportion increases from 20% to 40%, the BCAFL framework does not experience performance degradation due to the intensified asynchronous interference. Conversely, facing the increased pressure of network heterogeneity, the delay-aware weight $e^{-\mu (t-\tau _i)}$ within the dynamic aggregation factor adaptively reduces the aggregation proportion of stale gradients. This enables the convergence curve of the framework under the 40% delay proportion on the CIFAR10 dataset to exhibit even better convergence stability than in the 20% scenario.

### Cache threshold analysis

**Fig. 9:**
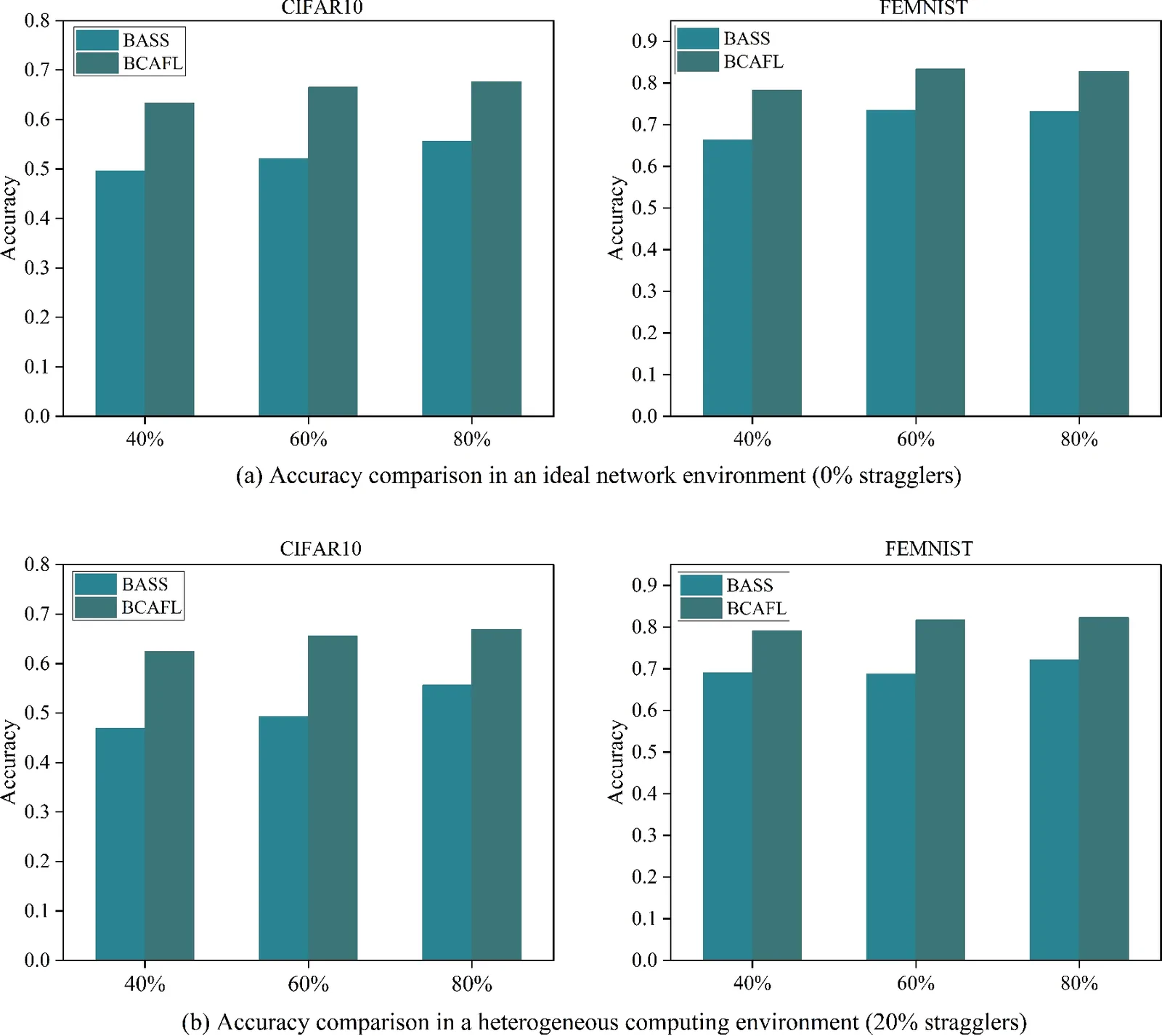
Accuracy comparison under different network environments and cache thresholds.

The global aggregation cache threshold determines the proportion of local models required to be collected for a single update round. To evaluate the impact of this parameter on convergence accuracy, experiments were conducted to test the accuracy performance of BCAFL and BASS on the CIFAR10 and FEMNIST datasets under both a synchronous network without straggler nodes and a heterogeneous environment containing 20% delayed nodes. The results are illustrated in Fig. 9.

Figure 9a presents the test results in the synchronous network. Within the cache threshold range of 40% to 80%, the convergence accuracy of BCAFL is consistently higher than that of BASS. Compared to the SignSGD mechanism of BASS, BCAFL combines PowerSGD and MAML to update the local model, preserving the gradient structure and data features while compressing the communication overhead. Furthermore, it utilizes the MIDA mechanism for anomaly filtering and dynamic aggregation, thereby reducing aggregation errors and enhancing the convergence accuracy of the framework across different thresholds. Figure 9b further illustrates the test results in a heterogeneous environment introducing 20% delayed clients. Facing the asynchronous interference caused by model upload lags, BCAFL consistently maintains an accuracy lead over BASS under all tested threshold configurations. Even in practical operating environments with some slow clients, the framework can preserve high model performance across diverse cache thresholds.

**Table 4:** The size of the model uploaded during one iteration of training (KB).

Dataset	Without PowerSGD	With PowerSGD
CIFAR10	764.60	77.74
FEMNIST	3399.74	26.67

### Communication efficiency and convergence accuracy

To quantitatively evaluate the communication efficiency of the BCAFL framework, this section employs the per-round communication payload and the model convergence accuracy under different compression configurations as evaluation metrics. The experiment first compares the per-round data transmission volume of the framework without and with the PowerSGD compression mechanism, and subsequently compares the communication payload and convergence accuracy between BCAFL and BASS under different compression configurations.

As shown in Table 4, when the compression mechanism is not adopted, due to the necessity of transmitting the full model parameters, the per-round uploaded gradient sizes in the CIFAR10 and FEMNIST tasks reach 764.60 KB and 3399.74 KB, respectively. This is prone to trigger communication latency and congestion in bandwidth-constrained networks. In contrast, BCAFL integrated with the PowerSGD compression mechanism under the default configuration reduces the per-round transmission data volume for CIFAR10 to 77.74 KB, shrinking the data interaction scale by 89.83%. For the FEMNIST task with higher parameter dimensions, its transmission size is 26.67 KB, achieving a parameter compression ratio of 99.22%. Experimental data indicate that this compression mechanism can effectively alleviate communication bottlenecks, reducing the communication overhead of high-dimensional parameter interactions while maintaining the convergence accuracy of the global model.

**Fig. 10:**
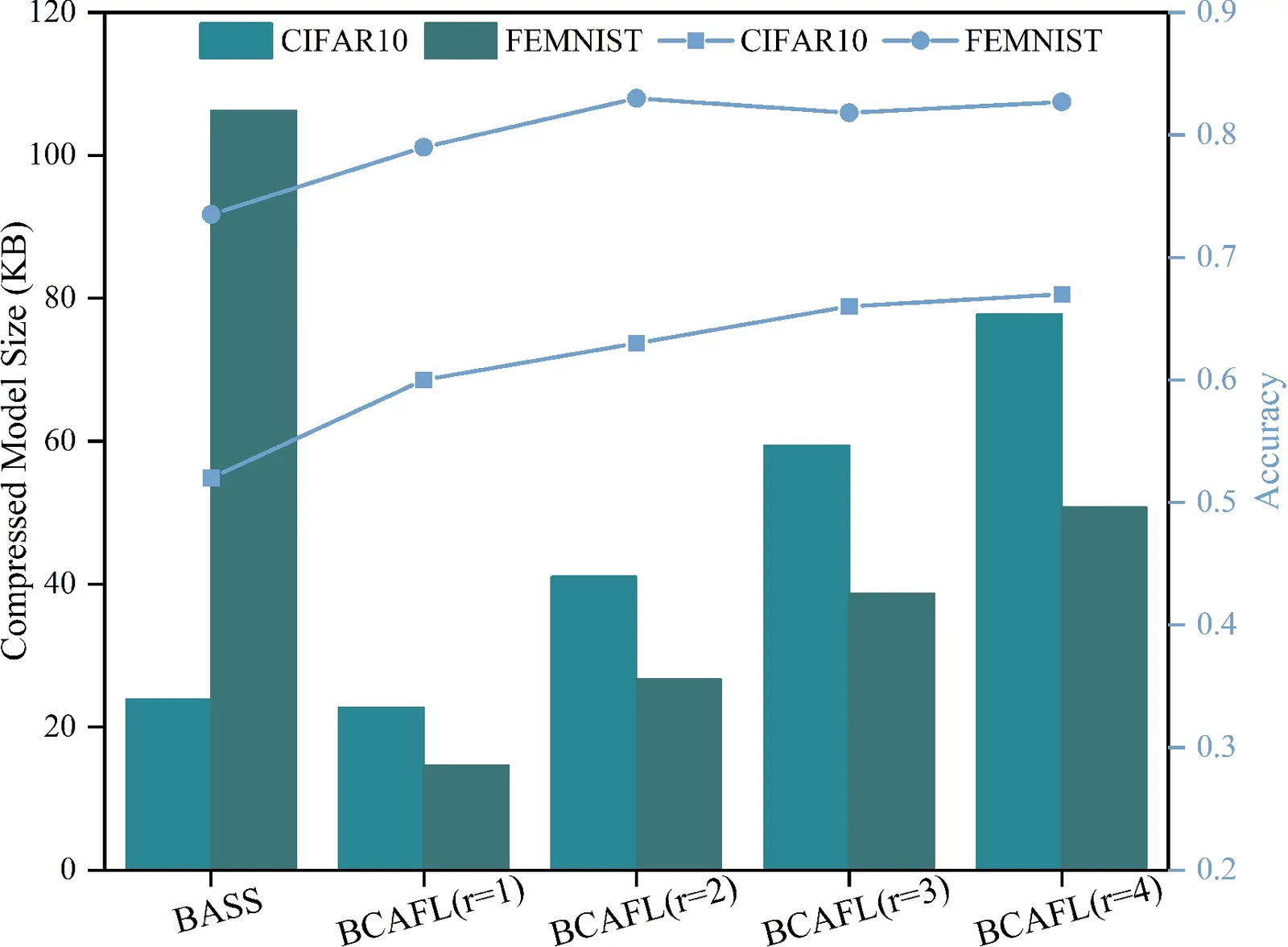
Evaluation of transmission payload and convergence accuracy across different compression schemes.

**Fig. 11:**
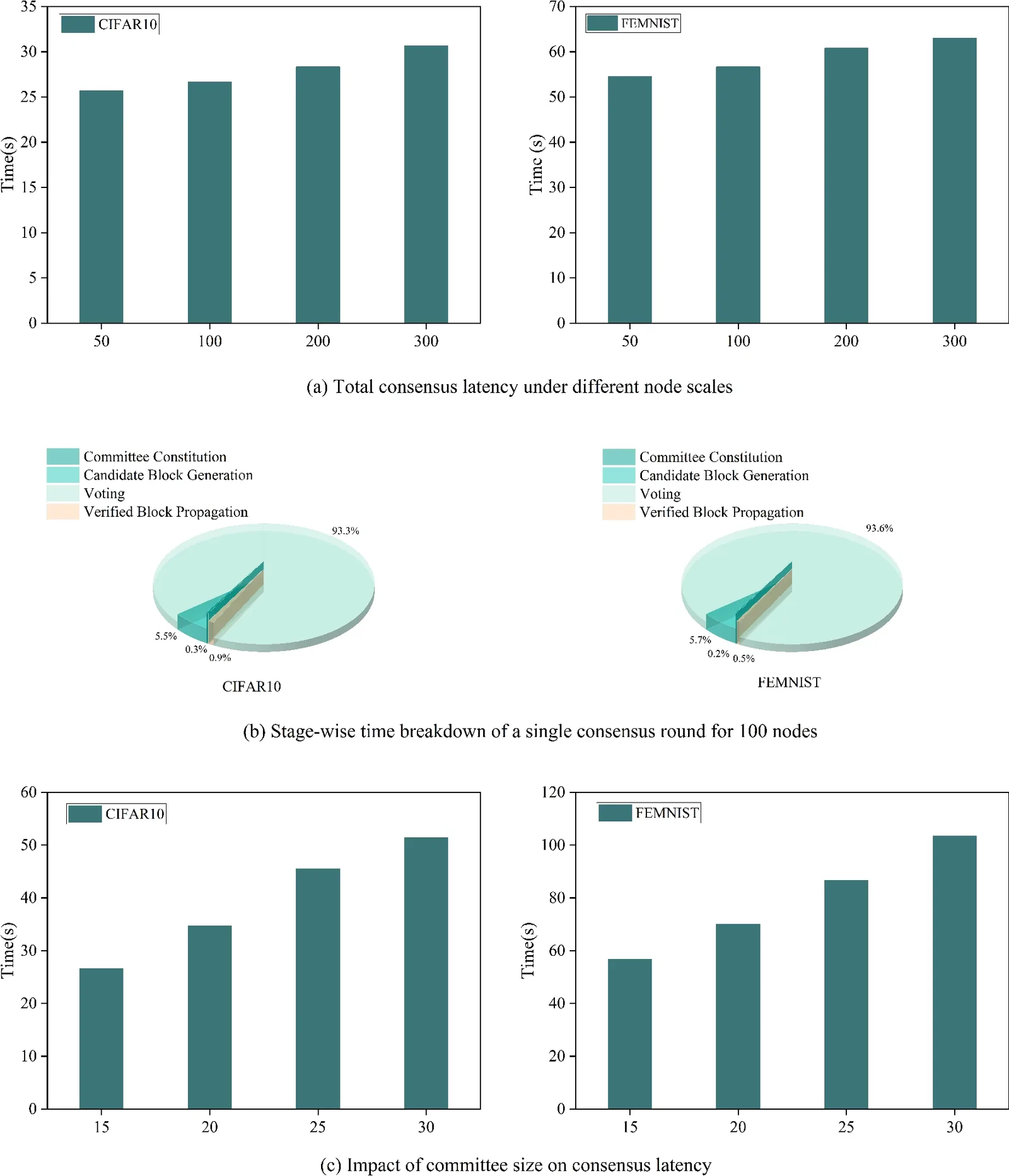
Time overhead of the BCAFL consensus mechanism.

Figure 10 further compares the performance of BCAFL and BASS regarding per-round communication payload and convergence accuracy. In the CIFAR10 task, BCAFL achieves a 97.03% compression rate under the *r = 1* configuration, outperforming the BASS scheme with a compression rate of 96.87% in both communication payload and convergence accuracy. Meanwhile, BCAFL supports further enhancement of the model convergence accuracy by adjusting the rank parameter ($r \in \{2, 3, 4\}$, corresponding to compression rates from 94.63% to 89.83%). In the FEMNIST task, the compression rates of BCAFL under $r \in \{1, 2, 3, 4\}$ are maintained between 98.51% and 99.57%, and its per-round transmission data volume and test accuracy both surpass those of BASS. The sign quantization mechanism adopted by BASS introduces quantization errors by discarding gradient magnitude information, and its fixed quantization bit-width makes it difficult to flexibly adjust the communication overhead according to actual network conditions. In contrast, BCAFL employs a low-rank matrix approximation strategy to perform dimensionality reduction and compression on local gradients, which more accurately preserves the descent direction of the original gradients while effectively reducing the transmission data volume. Furthermore, as demonstrated by the aforementioned experimental results, BCAFL supports achieving a dynamic balance between communication payload and model accuracy by tuning the rank parameter *r*.

**Table 5:** Comparison of cumulative on-chain storage overhead for different schemes (MB).

Round	CIFAR10		FEMNIST	
	BCAFL	BCAFL (No IPFS)	BCAFL	BCAFL (No IPFS)
50	0.582	77.16	0.582	339.65
100	1.157	152.8	1.156	672.63
150	1.728	228.46	1.729	1005.61
200	2.263	304.10	2.264	1338.60

### System overhead and scalability assessment

To quantitatively evaluate the system overhead and scalability of the BCAFL framework, this section employs the cumulative on-chain storage overhead, along with the consensus latency, transaction throughput, computational resource utilization, and energy consumption varying with the network scale, as evaluation metrics. The experiment first compares the cumulative on-chain storage requirements of different schemes, and subsequently quantitatively analyzes the consensus performance and system resource consumption of the framework under a network scale expansion environment.

Regarding the storage overhead, Table 5 summarizes the cumulative on-chain storage requirements of BCAFL and the direct on-chain full model scheme BCAFL ﻿(No IPFS) under different training rounds. The results demonstrate that the on-chain storage consumption of BCAFL (No IPFS) grows linearly with the iteration rounds, reaching 1338.60 MB on the FEMNIST dataset at the $200^{\text {th}}$ round. In contrast, BCAFL stores high-dimensional model data via IPFS, and the blockchain mainnet only records the CID, resulting in a total storage consumption of approximately 2.26 MB for 200 rounds. This result indicates that the IPFS off-chain storage mechanism effectively controls the block bloat caused by continuous iterations, guaranteeing the long-term operational capability of the framework.

Based on verifying that the storage overhead is controllable, we further evaluate the consensus performance of the framework under node scale expansion. By varying the total number of blockchain nodes under the condition of a fixed committee size of *K = 15*, the time consumption of each phase in a single consensus round and the impact of committee size variations on the total time consumption were respectively measured in a 100-node network environment. The relevant results are shown in Fig. 11. Among them, Fig. 11a illustrates the impact of an increasing number of blockchain nodes on the block final confirmation and distribution latency. Because the fixed-size committee mechanism controls the consensus verification overhead, and recording only lightweight IPFS content identifiers on-chain avoids the broadcast latency triggered by high-dimensional parameters, the combination of both guarantees the high efficiency of the framework during node scale expansion. To perform an in-depth analysis of the performance overhead, the experiment measured the specific time consumption of each phase of a single consensus round in a 100-node environment. As shown in Fig. 11b, the complete process comprises committee constitution, candidate block generation, voting, and verified block propagation, among which the voting phase constitutes the primary source of performance overhead. Because the execution efficiency of this phase is influenced by the number of committee members, this section further evaluates the variation trend of total consensus latency with the committee size. As illustrated in Fig. 11c, the total block latency exhibits linear growth with the expansion of the committee size. Although a larger committee size can enhance block credibility and the fault tolerance upper bound of the framework through broader consensus, the communication and computational interaction overhead required for nodes to reach an agreement also rises accordingly. This trade-off underscores the necessity of achieving a balance between security boundaries and verification efficiency.

To quantitatively evaluate the system resource consumption of the framework as the network scale expands, the experiment further records the transaction throughput, computational cost, and energy consumption under varying node scales. The specific definitions and calculation methods for each metric are as follows:

- **Transaction throughput:** Refers to the number of update transactions processed per unit of time during the consensus phase. It is calculated by dividing the total number of transactions that complete signature verification and are packed into the confirmed block within a single consensus cycle by the consensus latency of that cycle.
- **Computational cost:** Quantified by the average utilization rate of hardware resources within a single global iteration round. The experiment samples the instantaneous CPU and GPU utilization rates at a period of 500 ms. After a single iteration round concludes, the average utilization rates for this round, $U_{CPU}$ and $U_{GPU}$, are obtained by calculating the average of all sampling points within the round.
- **Energy consumption:** Refers to the cumulative energy consumption of a single global iteration round. Its calculated value is the product of the total time consumption of a single global iteration round and the average total power of the system. Specifically, the average GPU power is derived by averaging the instantaneous power collected via the hardware interface (RTX 3080 Ti, TDP 350W); the CPU power is estimated using a linear power consumption model, multiplying the Thermal Design Power (Intel Xeon Silver 4214R, TDP 100W) by the average utilization rate $U_{CPU}$ for the current round.

**Table 6:** Comprehensive analysis of consensus overhead under varying network sizes.

Network size	Dataset	Transaction throughput (TPS)	Computation cost (CPU/GPU)	Overhead energy (kJ)
N = 50	CIFAR10	0.3609	26.3%/33.1%	7.82
	FEMNIST	0.1774	16.1%/4.2%	9.06
N = 100	CIFAR10	0.3538	26.6%/33.2%	7.87
	FEMNIST	0.1756	16.2%/4.4%	9.12
N = 200	CIFAR10	0.3486	28.6%/33.2%	8.05
	FEMNIST	0.1737	17.9%/4.5%	9.27
N = 300	CIFAR10	0.3259	29.4%/30.6%	8.15
	FEMNIST	0.1681	18.5%/5.3%	9.51

The experimental results are presented in Table 6. As the network node scale expands from *N = 50* to *N = 300*, the transaction throughput of the system decreases by approximately 9.7% and 5.2% on the CIFAR10 and FEMNIST datasets, respectively. This is primarily influenced by the block broadcast and network communication latency subsequent to the node increase. Regarding computational cost and energy consumption, considering that this experiment employs a single-machine multi-node simulation environment, the rise in average CPU utilization is primarily dedicated to processing the growing signature verification and P2P network communication requests as the network scale expands; the average GPU utilization remains relatively stable across different scales, and the total energy consumption of a single simulation round only exhibits a marginal increase of 4% to 5%. The core of this stable resource overhead performance lies in the fact that BCAFL achieves a logical decoupling of the consensus computational overhead from the total number of nodes in the entire network via the VRF mechanism. When expanding the network scale, high-frequency aggregation computations are restricted within the fixed committee, and the vast majority of newly added nodes only need to execute low-overhead block state synchronization. The aforementioned metrics indicate that the computational and communication complexity of this framework does not experience severe bloat with the expansion of the total network scale, thereby possessing excellent logical scalability while maintaining a reasonable throughput.

## Discussions and limitations

The experimental evaluation in this paper validates the effectiveness of BCAFL under the current assumptions and simulation scale; however, the framework still exhibits limitations in practical deployment and theoretical research. First, regarding the compatibility between privacy protection and the anomaly detection mechanism, the framework currently does not introduce cryptographic defense measures, such as differential privacy or homomorphic encryption, against feature inference attacks. Because the MIDA mechanism designed in BCAFL relies on the mutual information between local updates to evaluate statistical consistency for implementing anomaly filtering, directly introducing noise-addition techniques like differential privacy would alter the original distribution of model parameters. This would interfere with the accuracy of the mutual information metric, thereby reducing the system’s effectiveness in identifying poisoning attacks. Therefore, how to introduce privacy protection measures while maintaining anti-poisoning robustness, in order to balance the performance trade-off between anomaly detection and privacy defense, is a question that needs to be explored in future research.

Second, the adaptability of the framework in heterogeneous network environments and the theoretical soundness of the incentive mechanism still require improvement. On the one hand, the semi-asynchronous aggregation mechanism of BCAFL relies on a statically preset cache threshold to trigger global updates. In practical operating environments characterized by frequent fluctuations in the online status of nodes or significant variances in network latency, a fixed threshold configuration lacks adaptive adjustment capabilities. This may lead to additional blocking delays in certain rounds, thereby degrading aggregation efficiency. On the other hand, although experimental results indicate that the dynamic stake-based mechanism can reduce the weight of malicious nodes, this reward and penalty model has not yet provided a formal game-theoretic analysis. Considering the long-term concealment or dynamic collusion strategies that rational nodes might adopt in a decentralized system, future work needs to establish relevant game-theoretic models to formally define the incentive compatibility and long-term stability of this mechanism, and further explore adaptive aggregation control strategies based on real-time network status feedback.

## Conclusion

This paper proposes BCAFL, a blockchain framework for semi-asynchronous federated learning. The framework utilizes a semi-asynchronous mechanism to reduce node waiting latency in heterogeneous computing environments, introduces MAML and PowerSGD to enhance the model’s local adaptation capability under non-independent and identically distributed (non-IID) data while reducing communication overhead, and integrates IPFS to alleviate the on-chain storage load. Meanwhile, the framework designs a dynamic aggregation mechanism based on mutual information and delay awareness, as well as a consensus protocol based on dynamic stake, to defend against poisoning and Byzantine attacks. Experimental results demonstrate that under the configured scenarios where the proportion of Byzantine nodes in the entire network does not exceed 1/3 and non-colluding poisoning attacks are present, BCAFL can effectively filter poisoned parameters, resist consensus disruption, and guarantee the stable convergence of the global model.

Future research work will center around the limitations described in the DISCUSSIONS AND LIMITATIONS section, with the primary focus including exploring privacy protection methods that accommodate anomaly detection, developing adaptive aggregation control strategies based on network state feedback, and establishing formal game-theoretic models for the incentive mechanism.

## Data Availability

The datasets analysed during the current study are available in the public open-source repositories. Specifically, the datasets can be accessed at the following persistent web links: LEAF (FEMNIST): https://leaf.cmu.edu/ CIFAR10: https: //www.cs.toronto.edu/kriz/cifar.html

## References

[1] Nguyen, D. C. et al. Federated learning for Internet of Things: A comprehensive survey. *IEEE Commun. Surv. Tut.* **23**, 1622–1658 (2021).

[2] McMahan, B., Moore, E., Ramage, D., Hampson, S. & Agüera y Arcas, B. Communication-efficient learning of deep networks from decentralized data. In: *Proc. Mach. Learn. Res.*, vol. 54, 1273–1282 (2017).

[3] Li, T., Sahu, A. K., Talwalkar, A. & Smith, V. Federated learning: Challenges, methods, and future directions. *IEEE Signal Process. Mag.* **37**, 50–60 (2020).

[4] Feng, Y. et al. A survey of security threats in federated learning. *Complex Intell. Syst.* **11**, 165 (2025).

[5] Bagdasaryan, E., Veit, A., Hua, Y., Estrin, D. & Shmatikov, V. How to backdoor federated learning. *Proc. Mach. Learn. Res.* **108**, 2938–2948 (2020).

[6] Yao, W., Zhou, T., Han, Y. & Wang, X. Verifiable secure aggregation scheme for privacy protection in federated learning networks. *Discov. Comput.* **28**, 175 (2025).

[7] Mahmud, F., Mahmud, F. & Rahman, R. M. A survey of federated learning: Advances in architecture, synchronization, and security threats. *Comput. Mater. Contin.* **86** (2026). 10.32604/cmc.2025.073519.

[8] Orabi, M. M., Emam, O. & Fahmy, H. Adapting security and decentralized knowledge enhancement in federated learning using blockchain technology: literature review. *J. Big Data* **12**, 55 (2025).

[9] Qu, Y., Pokhrel, S. R., Garg, S., Gao, L. & Xiang, Y. A blockchained federated learning framework for cognitive computing in Industry 4.0 networks. *IEEE Trans. Ind. Inform.* **17**, 2964–2973 (2021).

[10] Xu, M. et al. SPDL: A blockchain-enabled secure and privacy-preserving decentralized learning system. *IEEE Trans. Comput.* **72**, 548–558 (2023).

[11] Zangoti, H., Makki, A. P., Khan, W. Z. & Pissinou, N. Enhancing blockchain storage efficiency in mobile IoT through multidimensional structures and collective signing. In: *Proc. IEEE Int. Conf. Blockchain*, 478–483, 10.1109/Blockchain62396.2024.00070 (2024).

[12] Xu, C. et al. BASS: A blockchain-based asynchronous SignSGD architecture for efficient and secure federated learning. *IEEE Trans. Dependable Secur. Comput.* **21**, 5388–5402 (2024).

[13] Liang, J., Li, S., Cao, B., Jiang, W. & He, C. OmniLytics: A blockchain-based secure data market for decentralized machine learning. Preprint at arXiv:2107.05252 (2021).

[14] Zhou, S., Wang, Y., Wang, L., Chen, L. & Wang, Y. FedDG: An efficient federated learning framework for IoT communication based on dynamic fusion and gradient compression. *IEEE Trans. Ind. Inform.* **22**, 2530–2540 (2026).

[15] Bernstein, J., Wang, Y.-X., Azizzadenesheli, K. & Anandkumar, A. signSGD: Compressed optimisation for non-convex problems. *Proc. Mach. Learn. Res.* **80**, 560–569 (2018).

[16] Jin, R. et al. Sign-based gradient descent with heterogeneous data: Convergence and Byzantine resilience. *IEEE Trans. Neural Netw. Learn. Syst.* **36**, 3834–3846 (2025). 38215315 10.1109/TNNLS.2023.3345367

[17] Vogels, T., Karimireddy, S. P. & Jaggi, M. PowerSGD: Practical low-rank gradient compression for distributed optimization. In: *Adv. Neural Inf. Process. Syst.*, vol. 32 (2019).

[18] Fallah, A., Mokhtari, A. & Ozdaglar, A. Personalized federated learning with theoretical guarantees: A model-agnostic meta-learning approach. In: *Adv. Neural Inf. Process. Syst.*, vol. 33, 3557–3568 (2020).

[19] Shayan, M., Fung, C., Yoon, C. J. M. & Beschastnikh, I. Biscotti: A blockchain system for private and secure federated learning. *IEEE Trans. Parallel Distrib. Syst.* **32**, 1513–1525 (2021).

[20] Kalapaaking, A. P. et al. Auditable and verifiable federated learning based on blockchain-enabled decentralization. *IEEE Trans. Neural Netw. Learn. Syst.* **36**, 102–115 (2025). 38875094 10.1109/TNNLS.2024.3407670

[21] Mugunthan, V., Rahman, R. & Kagal, L. BlockFLow: An accountable and privacy-preserving solution for federated learning. Preprint at arXiv:2007.03856 (2020).

[22] Xu, M. et al. SPDL: A blockchain-enabled secure and privacy-preserving decentralized learning system. *IEEE Trans. Comput.* **72**, 548–558 (2023).

[23] Chen, X., Feng, C. & Wang, S. AIDFL: An information-driven anomaly detector for data poisoning in decentralized federated learning. *IEEE Access* **13**, 50017–50031 (2025).

[24] Zhou, Z. et al. Blockchain and signcryption enabled asynchronous federated learning framework in fog computing. *Digit. Commun. Netw.* **11**, 442–454 (2025).

[25] Uddin, M. P., Xiang, Y., Lu, X., Yearwood, J. & Gao, L. Mutual information driven federated learning. *IEEE Trans. Parallel Distrib. Syst.* **32**, 1526–1538 (2021).

[26] Zhang, S. DAG-AFL: Directed acyclic graph-based asynchronous federated learning. In: *2025 IEEE International Conference on Multimedia and Expo (ICME)* 1–6 (IEEE, 2025).

[27] Huang, X., Deng, X., Chen, Q. & Zhang, J. AFLChain: Blockchain-enabled asynchronous federated learning in edge computing network. In: *2023 IEEE 97th Vehicular Technology Conference (VTC2023-Spring)*, 1–5 (IEEE, 2023).

[28] Jin, R., Hu, J., Min, G. & Mills, J. Lightweight blockchain-empowered secure and efficient federated edge learning. *IEEE Trans. Comput.* **72**, 3314–3325 (2023).

[29] Du, Y., Wang, Z., Leung, C. & Leung, V. C. M. Accelerating and securing blockchain-enabled distributed machine learning. *IEEE Trans. Mob. Comput.* **23**, 6712–6730 (2024).

[30] Liu, X. et al. Group BFT: Two-round BFT protocols via replica grouping. *IEEE Trans. Dependable Secur. Comput.* **22**, 6309–6326 (2025).

[31] Chung, H.-T., Cheng, S.-H., Chen, Y.-J. & Wang, L.-C. BZ-BFT: An efficient and scalable consensus mechanism for blockchain federated learning. In: *Proc. 2025 IEEE Wireless Commun. Netw. Conf. (WCNC)*, 1–6, 10.1109/WCNC61545.2025.10978754 (2025).

[32] Islam, M. M., Merlec, M. M. & IN, H. P. Proof of random leader: A fast and manipulation-resistant proof-of-authority consensus algorithm for permissioned blockchains using verifiable random function. *IEEE Trans. Serv. Comput.* **18**, 1655–1668 (2025).

[33] Caldas, S. et al. LEAF: A benchmark for federated settings. Preprint at arXiv:1812.01097 (2018).

[34] Li, A. et al. LotteryFL: Personalized and communication-efficient federated learning with lottery ticket hypothesis on non-iid datasets. Preprint at arXiv:2008.03371 (2020).

[35] He, K., Zhang, X., Ren, S. & Sun, J. Deep residual learning for image recognition. In: *Proc. IEEE Conf. Comput. Vis. Pattern Recognit.*, 770–778 (2016).

[36] Xie, C., Koyejo, S. & Gupta, I. Asynchronous federated optimization. Preprint at arXiv:1903.03934 (2019).

[37] Blanchard, P., El Mhamdi, E. M., Guerraoui, R. & Stainer, J. Machine learning with adversaries: Byzantine tolerant gradient descent. In: *Adv. Neural Inf. Process. Syst.*, vol. 30 (2017).

[38] Fung, C., Yoon, C. J. M. & Beschastnikh, I. Mitigating sybils in federated learning poisoning. Preprint at https://arxiv.org/abs/1808.04866 (2018).

[39] Karimireddy, S. P., He, L. & Jaggi, M. Learning from history for Byzantine robust optimization. In: *Proc. 38th Int. Conf. Mach. Learn. (ICML)*, 5311–5319 (2021).

